# Nitrite Derived from Endogenous Bacterial Nitric Oxide Synthase Activity Promotes Aerobic Respiration

**DOI:** 10.1128/mBio.00887-17

**Published:** 2017-08-01

**Authors:** Sujata S. Chaudhari, Minji Kim, Shulei Lei, Fareha Razvi, Abdulelah A. Alqarzaee, Elizabeth H. Hutfless, Robert Powers, Matthew C. Zimmerman, Paul D. Fey, Vinai C. Thomas

**Affiliations:** a Center for Staphylococcal Research, Department of Pathology and Microbiology, University of Nebraska Medical Center, Omaha, Nebraska, USA; b Department of Chemistry, University of Nebraska, Lincoln, Lincoln, Nebraska, USA; c Cellular and Integrative Physiology, University of Nebraska Medical Center, Omaha, Nebraska, USA; University of Tubingen; Harvard Medical School

**Keywords:** *Staphylococcus aureus*, TCA cycle, cytochrome *bo* oxidase, nitric oxide synthase, nitrite, quinol oxidase, respiration

## Abstract

Macrophage-derived nitric oxide (NO·) is a crucial effector against invading pathogens. Yet, paradoxically, several bacterial species, including some pathogens, are known to endogenously produce NO· via nitric oxide synthase (NOS) activity, despite its apparent cytotoxicity. Here, we reveal a conserved role for bacterial NOS in activating aerobic respiration. We demonstrate that nitrite generated from endogenous NO· decomposition stimulates quinol oxidase activity in *Staphylococcus aureus* and increases the rate of cellular respiration. This not only supports optimal growth of this organism but also prevents a dysbalance in central metabolism. Further, we also show that activity of the SrrAB two-component system alleviates the physiological defects of the *nos* mutant. Our findings suggest that NOS and SrrAB constitute two distinct but functionally redundant routes for controlling staphylococcal respiration during aerobic growth.

## IMPORTANCE

Despite its potential autotoxic effects, several bacterial species, including pathogenic staphylococcal species, produce NO· endogenously through nitric oxide synthase (NOS) activity. Therefore, how endogenous NO· influences bacterial fitness remains unclear. Here we show that the oxidation of NO· to nitrite increases aerobic respiration and consequently optimizes central metabolism to favor growth. Importantly, we also demonstrate that cells have a “fail-safe” mechanism that can maintain respiratory activity through the SrrAB two-component signaling regulon should NOS-derived nitrite levels decrease. These findings identify NOS and SrrAB as critical determinants of staphylococcal respiratory control and highlight their potential as therapeutic targets.

## INTRODUCTION

As an arginine auxotroph, *Staphylococcus aureus* must primarily rely on efficient arginine uptake and utilization mechanisms for optimal colonization and pathogenesis in the host (1). It is, then, no surprise that *S. aureus* employs three pathways to rapidly catabolize arginine upon its entry into cells. The first pathway involves proteins encoded by the arginine deiminase (ADI) operon that converts arginine to citrulline and produces ammonia in the process (2). Notably, the predominant community-associated methicillin-resistant *S. aureus* (CA-MRSA) isolates of the USA300 lineage have acquired an additional copy of the ADI pathway in the arginine catabolic mobile element (ACME), a genetic determinant that has been linked to its overwhelming success as a pathogen (3). The second metabolic route makes use of the enzyme arginase that produces ornithine and urea from arginine. Urea is further converted to ammonia with the aid of urease. Both the ADI and arginase pathways are thought to play important roles under acidic conditions, as ammonia resulting from these pathways helps to maintain pH homeostasis (2, 3). Additionally, activity of the ADI pathway is also important under anaerobic conditions, as it can be a significant source of cellular ATP (2). The enzyme nitric oxide synthase (NOS), which converts arginine to citrulline and nitric oxide, constitutes the third route for arginine catabolism. However, understanding of NOS function in staphylococcal physiology remains incomplete, particularly since the by-product of this pathway, NO·, can be cytotoxic to bacteria.

Multiple studies have demonstrated that endogenous NO· resulting from NOS activity constitutes a defense against reactive oxygen species (ROS) (4–6). Potential mechanisms include the ability of NO· to limit recycling of Fe^2+^ following nitration of cellular thiols, which otherwise would lead to macromolecular damage from hydroxyl radicals generated by Fenton chemistry, and transcriptional activation of catalase by NO· (4). More recently, staphylococcal NOS was suggested to play a role in adaptation to microaerobic environments (7). Accordingly, *S. aureus* was shown to enzymatically convert endogenous NO· to nitrate and utilize it as a substrate for respiration via nitrate reductase under conditions of oxygen limitation (7). However, given the pleiotropic effects of NO· on cell physiology, alternative functions for bacterial NOS cannot be ruled out. In higher eukaryotes, NO· also acts as a diffusible signal that mediates vascular relaxation in addition to executing its functions associated with host defense (8). As a signal, NO· interacts with the heme prosthetic group of soluble guanylyl cyclase (sGC) and triggers catalytic activity of sGC, resulting in the synthesis of the second messenger, cyclic GMP (cGMP). Muscle relaxation immediately follows cGMP signaling (8). Interestingly, various heme-based NO· sensor domain (H-NOX)-containing proteins such as eukaryotic sGC are also present in bacterial species and can control c-di-GMP levels to modulate various physiologic processes (9). Despite these broad similarities, reports that suggest whether NO· itself can act as a signaling molecule in bacteria are scarce (9). In addition to these proposed functions, NO· is also rapidly converted to more stable metabolites such as nitrite and nitrate under aerobic conditions and a role for these metabolites in NO·-dependent processes has not been rigorously pursued.

Due to its high reactivity, short biological half-life, and multiple potential targets *in vivo*, we hypothesized that NOS-derived NO· or its stable derivatives may affect critical physiological functions in bacteria. In this study, we demonstrated that NOS unexpectedly activates quinol oxidase-dependent cellular respiration and regulates bacterial growth through nitrite, a product of NO· decomposition. *S. aureus* mutants that lacked NOS activity displayed a diminished ability to respire, especially when SrrAB-dependent cellular respiration was compromised. Given recent efforts to use bacterial NOS as a therapeutic target against *S. aureus* (10), our findings assume critical importance as we not only identify the physiological significance of NOS in bacterial respiration but also shed light on alternative respiratory control mechanisms that stabilize cell physiology in the absence of NOS.

## RESULTS

### Mutation of *nos* delays post-exponential-phase growth.

**FIG 1:**
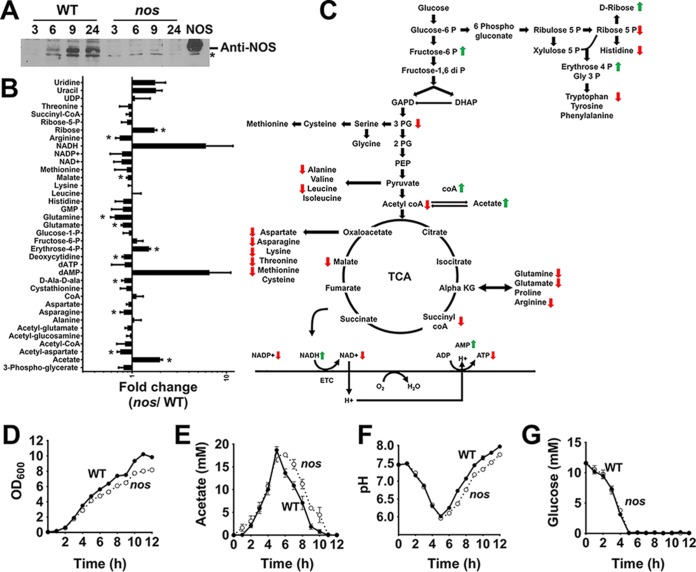
Inactivation of *nos* affects central metabolism. (A) Post-exponential-phase expression of NOS (~42 kDa) was detected by Western blot analysis using polyclonal antibodies raised against recombinant NOS. Fifty micrograms of total protein from cell lysates corresponding to various stages of growth was loaded in each lane. The rightmost lane contains recombinant NOS (100 ng). *, nonspecific protein band. (B) 2D-NMR spectroscopic analysis of *nos* mutant following supplementation of media with ^13^C_6_-labeled glucose (means ± standard errors of the means [SEM], *n* = 3, paired Student's *t* test; *, *P* < 0.05). (C) Schematic representation of the relative changes of intracellular metabolites in the *nos* mutant compared to the wild-type strain. Green and red arrows represent respective increases and decreases in metabolite concentrations in the *nos* mutant relative to the wild-type strain. (D to G) Growth (D), acetate (E), pH (F), and glucose (G) kinetics of the wild-type (WT) strain and *nos* mutant were monitored under conditions of aerobic growth at 37°C for 12 h (means ± SEM, *n* = 3).

To gain better insight into the function of NOS, we initially assessed its expression during different stages of *S. aureus* growth using NOS-specific polyclonal antibodies. Grown aerobically in tryptic soy broth (TSB; 14 mM glucose), NOS was predominantly expressed at the post-exponential and stationary phases when glucose was mostly depleted from the media (Fig. 1A). Although this is not entirely surprising, since arginine transport into cells is inhibited by the presence of excess glucose during exponential growth, it does suggest a potential role for NOS in post-exponential-phase metabolism. To test this hypothesis, we followed post-exponential-phase metabolic changes within the wild-type (WT) strain (*S. aureus* JE2) and its isogenic *nos* mutant by two-dimensional (2D) ^1^H-^13^C heteronuclear single quantum coherence (HSQC) nuclear magnetic resonance (NMR) spectroscopy after supplementing media with ^13^C_6_-labeled glucose. Prominent metabolic differences in the *nos* mutant included decreased levels of tricarboxylic acid (TCA) cycle intermediates and amino acids generated from those intermediates (Fig. 1B and C; see also Fig. S1 and Text S1 in the supplemental material), which suggested that TCA cycle activity may be decreased in the *nos* mutant relative to the wild-type strain. Consistent with this interpretation, we also observed an increase in levels of pentose phosphate intermediates such as d-ribose and erythrose-4 phosphate (Fig. 1B and C) whose intracellular levels characteristically increase following arrest of TCA cycle activity (11). To validate the effects of NOS on TCA cycle activity, we carefully monitored growth, pH, and acetate consumption at the post-exponential phase. It is well established that post-exponential-phase growth of *S. aureus* is primarily dependent on the oxidation of acetate through the TCA cycle (12). Consistent with decreased TCA cycle activity, we observed a modest but significant delay in post-exponential-phase growth (Fig. 1D) and in the ability to consume extracellular acetate (Fig. 1E). This was also reflected in delayed alkalization of the media by the *nos* mutant (Fig. 1F). The growth delay exhibited by the *nos* mutant did not result from a decrease in overall glucose consumption, as the wild-type and mutant strains displayed similar kinetics of glucose uptake (Fig. 1G). Significantly, all these phenotypes could be complemented in the *nos* mutant by plasmid-based expression of the *nos* gene in *trans*, ruling out any polar effects associated with the *nos* mutation (Fig. S2).

10.1128/mBio.00887-17.1 TEXT S1  Supplemental figure legends. Download TEXT S1, DOCX file, 0.02 MB. Copyright © 2017 Chaudhari et al. 2017 Chaudhari et al. This content is distributed under the terms of the Creative Commons Attribution 4.0 International license.

10.1128/mBio.00887-17.2 FIG S1  Representative 2D 1H-13C HSQC NMR spectrum of intracellular metabolites. Download FIG S1, TIF file, 0.2 MB. Copyright © 2017 Chaudhari et al. 2017 Chaudhari et al. This content is distributed under the terms of the Creative Commons Attribution 4.0 International license.

10.1128/mBio.00887-17.3 FIG S2  Complementation of the *nos* mutant. Download FIG S2, TIF file, 1.2 MB. Copyright © 2017 Chaudhari et al. 2017 Chaudhari et al. This content is distributed under the terms of the Creative Commons Attribution 4.0 International license.

**FIG 2:**
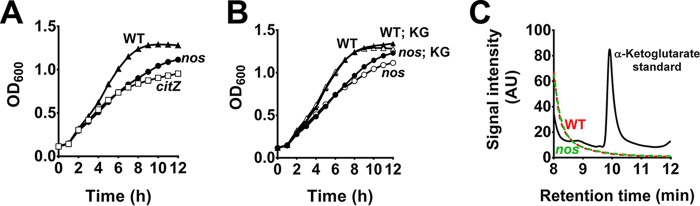
The growth defect of the *nos* mutant does not result from depletion of intracellular α-ketoglutarate. (A and B) Growth rates of *S. aureus* wild-type and mutant strains were monitored for 12 h in the absence (A) or presence (B) of 25 mM α-ketoglutarate (means ± SEM, *n* = 4). (C) α-Ketoglutarate levels were determined from culture supernatants of wild-type (WT) and mutant strains by HPLC (representative trace, *n* = 2). AU, arbitrary units.

Since *S. aureus* lacks the glyoxalate cycle that replenishes TCA cycle intermediates, consumption of two-carbon metabolites such as acetate following glucose exhaustion can effectively support post-exponential-phase growth only when amino acids are simultaneously utilized as a source of TCA cycle intermediates. Thus, it is plausible that NOS-mediated conversion of arginine to citrulline may eventually feed into the TCA cycle through the glutamate-α-ketoglutarate node and support acetate catabolism. Accordingly, the observed decrease in TCA cycle activity associated with the *nos* mutation could result from depletion of TCA cycle intermediates. Consistent with this hypothesis, NMR spectroscopic analysis suggested that glutamate and glutamine levels were lower in the *nos* mutant than in the wild-type strain (Fig. 1B and C). To test this hypothesis, we initially optimized conditions that would maximize the differences in growth of the *nos* mutant relative to the wild-type strain. It was observed that in TSB lacking glucose (diluted 50% [vol/vol]), growth of the *nos* mutant was delayed, even during the exponential phase, in a manner similar to that observed with a citrate synthase mutant, presumably due to a defective TCA cycle (Fig. 2A). We reasoned that supplementation of TCA cycle intermediates under these conditions would rescue the growth defect associated with the *nos* mutant. However, this was not evident, as supplementation of media with citrate (Fig. S3A), α-ketoglutarate (Fig. 2B), and succinate (Fig. S3C) did not restore growth of the *nos* mutant, despite complete consumption of these metabolites during growth (Fig. 2C; Fig. S3B and D). Further, the citrate synthase mutant was rescued using this approach by supplementing media with α-ketoglutarate, attesting to the functionality of this assay (Fig. S3E). These results suggest that although levels of TCA cycle metabolites were perturbed, the growth defect observed in the *nos* mutant did not result from these changes.

10.1128/mBio.00887-17.4 FIG S3  The growth defect of the *nos* mutant does not result from depletion of TCA cycle intermediates. Download FIG S3, TIF file, 0.4 MB. Copyright © 2017 Chaudhari et al. 2017 Chaudhari et al. This content is distributed under the terms of the Creative Commons Attribution 4.0 International license.

### NOS-dependent growth is associated with excreted nitrite.

**FIG 3:**
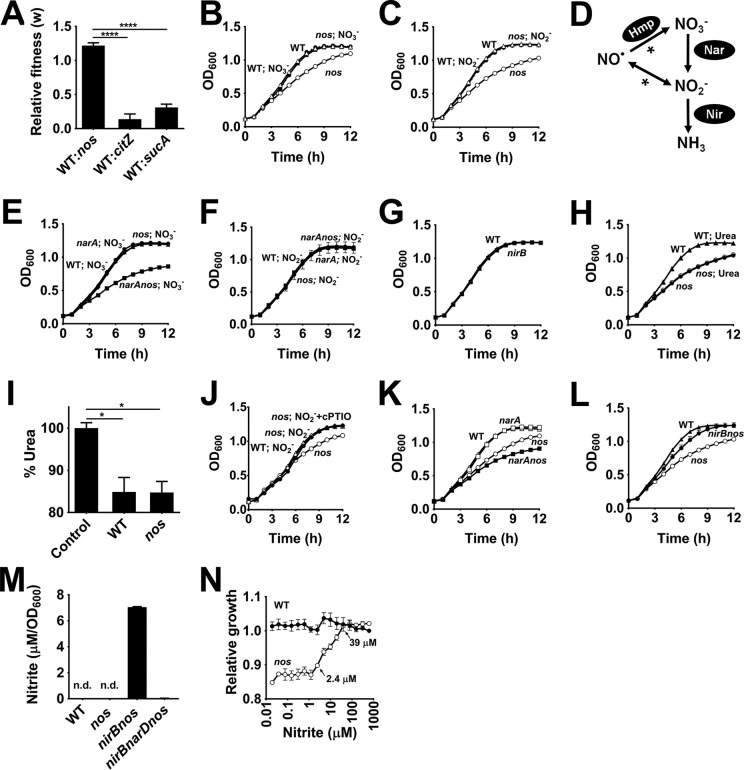
Nitrite derived from NOS activity enhances growth. (A) Competition of the wild-type and mutant strains following coculture in TSB (14 mM glucose) over a 24-h period (means ± SEM, *n* = 3, one-way ANOVA, Tukey’s posttest; ****, *P* < 0.00005). (B and C) Growth of wild-type (WT) strain and *nos* mutant following supplementation of media with either 0.5 mM nitrate (means ± SEM, *n* = 3) (B) or 0.5 mM nitrite (means ± SEM, *n* = 6) (C). (D) Schematic representing the fate of nitrate and nitrite derived from nitric oxide. While nitrate can be enzymatically derived from endogenous NO· by Hmp activity, it is also spontaneously (*) formed along with nitrites under aerobic conditions. Both nitrates and nitrites are eventually reduced to ammonia. (E and F) Growth of *narA nos* mutant following supplementation of media with 0.5 mM nitrate (means ± SEM, *n* = 4) (E) or 0.5 mM nitrite (means ± SEM, *n* = 4) (F). (G) Growth of *nirB* mutant relative to the wild-type (WT) strain (means ± SEM, *n* = 6). (H) Growth of wild-type (WT) strain and *nos* mutant following supplementation of media with 10 mM urea (means ± SEM, *n* = 9). (I) Consumption of urea by the wild-type (WT) strain and *nos* mutant following 24 h of growth (means ± SEM, *n* = 9, one-way ANOVA, Tukey’s posttest; *, *P* < 0.05). (J) Effect of cPTIO on nitrite-mediated restoration of growth in the *nos* mutant (means ± SEM, *n* = 3). (K and L) Growth of *narA nos* (mean ± SEM, *n* = 4) (K) and *nirB nos* (mean ± SEM, *n* = 6) (L) double mutants relative to the wild-type (WT) and *nos* mutant strains. (M) Nitrite levels were determined from culture supernatants after 24 h of growth using the Griess assay (means ± SEM, *n* = 6; n.d., undetectable levels). (N) The area under the growth curve (AUC) measuring growth of the wild-type strain and *nos* mutant at various concentrations (0.01 µM to 625 µM) of supplemented nitrite was compared to the AUC of the reference point (wild type, 625 µM nitrite) to obtain the minimum nitrite concentrations required to fully restore the growth defect associated with the *nos* mutant (means ± SEM, *n* = 3).

One consequence of decreased TCA cycle activity in the *nos* mutant would be reduced fitness of this strain relative to that of the wild-type strain. Surprisingly, competition assays where the WT strain and *nos* mutant were cocultured revealed increased fitness for the *nos* mutant, unlike the results seen with genuine TCA cycle-defective mutants (Fig. 3A; Fig. S4). This raised the possibility that unknown factors secreted by the wild-type strain could rescue defects associated with the *nos* mutant upon coculture. Since NOS-derived NO· is readily converted to nitrite and nitrate under aerobic conditions, both spontaneously and enzymatically, we hypothesized a role for these metabolites in enhancing growth of the *nos* mutant. Consistent with this hypothesis, addition of either nitrate (NO_3_^−^) or nitrite (NO_2_^−^) completely rescued the growth defect associated with the *nos* mutant (Fig. 3B and C).

10.1128/mBio.00887-17.5 FIG S4  Growth of *S. aureus* JE2 (WT) and *nos*, *sucA*, and *citZ* mutants. Download FIG S4, TIF file, 2.1 MB. Copyright © 2017 Chaudhari et al. 2017 Chaudhari et al. This content is distributed under the terms of the Creative Commons Attribution 4.0 International license.

It is possible that nitrate does not influence growth of the *nos* mutant *per se* but instead its reduction to nitrite by the respiratory nitrate reductase (encoded by the *narABCD* gene cluster) may be important (Fig. 3D). To test this hypothesis, we constructed a *narA nos* double mutant and grew it in the presence of nitrate. Unlike the *nos* mutant, whose growth was readily rescued by the addition of nitrate (NO_3_^−^), the *narA nos* double mutant did not exhibit growth rescue under the same conditions, thus excluding a role for nitrate as the modulator of growth (Fig. 3D and E). As expected, nitrite was effectively able to rescue the growth defect associated with the *narA nos* double mutant (Fig. 3F). However, since nitrite can be further converted to ammonia by the nitrite reductase (NirBD) cluster (Fig. 3D), we next tested whether it is nitrite or ammonia that aids growth of the *nos* mutant. We reasoned that if ammonia resulting from nitrite reductase activity is important for growth, a strain whose nitrite reductase complex is inactivated (*nirB* mutant) should phenocopy the growth characteristics of the *nos* mutant. However, the *nirB* mutant did not display any growth defect relative to the wild-type strain (Fig. 3G). Furthermore, addition of urea, a source of ammonia that would bypass the requirement of a functional *nirB*, did not rescue the growth defect in the *nos* mutant (Fig. 3H), despite a significant level of utilization of this metabolite by cells (Fig. 3I). Similarly, direct supplementation of ammonium salts (ammonium chloride) also did not rescue the growth defect associated with the *nos* mutant (Fig. S5A). Finally, it has been suggested that under certain circumstances, e.g., acidification, nitrite is converted back to NO· (Fig. 3D). However, addition of cPTIO [2-(4-carboxyphenyl)-4,4,5,5-tetramethylimidazoline-1-oxyl-3-oxide; cell-permeative NO· scavenger] did not reverse the nitrite-mediated growth rescue of the *nos* mutant (Fig. 3J). Collectively, these data suggest that nitrite alone acts as the primary modulator of growth in the *nos* mutant.

10.1128/mBio.00887-17.6 FIG S5  Growth analyses of JE2 (WT) and isogenic mutants. Download FIG S5, TIF file, 2 MB. Copyright © 2017 Chaudhari et al. 2017 Chaudhari et al. This content is distributed under the terms of the Creative Commons Attribution 4.0 International license.

Interestingly, whereas the *narA* single mutant displayed growth characteristics similar to those of the WT strain, the *narA nos* double mutant displayed an increased growth defect relative to the *nos* mutant (Fig. 3K). This was likely due to a decrease in nitrite levels following *narA* mutation rather than to a specific role for nitrate respiration, because nitrite supplementation could fully restore the growth defect associated with the *narA nos* double mutant (Fig. 3F). Further, inactivation of the nitrite reductase in the *nos* background mostly rescued the growth defect associated with *nos* mutation, as the extracellular concentration of nitrite increased in the *nirB nos* double mutant (Fig. 3L and M) due to a block in nitrite utilization. The source of nitrite in culture supernatants of the *nirB nos* mutant appear to result from small concentrations of nitrate present in TSB media. Consistent with this hypothesis, the *narD nirB nos* triple mutant did not significantly accumulate nitrite relative to the *nirB nos* mutant (Fig. 3M). Furthermore, the growth rescue observed in the *nirB nos* mutant was reversed in the *narD nirB nos* triple mutant to the levels observed in the *nos* mutant (Fig. S5B). Thus, in addition to underscoring the role of nitrite in mediating enhanced growth, these observations suggest that both nitrate and nitrite reductases are active at a basal level during aerobic respiration of *S. aureus* JE2 and that, at least, nitrate reductase activity partially offsets the growth defect of the *nos* mutant.

We next assessed the concentration of nitrite (NO_2_^−^) required to restore growth in the *nos* mutant. The effect of nitrite on bacterial growth was measured as a function of the area under the growth curve (AUC). The relative magnitude of growth (relative growth) of both the wild-type strain and the *nos* mutant were calculated from the ratio of the AUC of each sample to that of its corresponding control (in this case, the wild-type strain challenged with the highest concentration [625 µM] of nitrite) and displayed as a function of nitrite concentration. Titration of nitrite over a five-log concentration range (~0.01 µM to 1 mM) revealed that growth of the *nos* mutant could be fully rescued with as low as 39 µM nitrite (Fig. 3N). Importantly, our estimations of the levels of nitrite excreted over a period of 24 h by the *nirB* mutant (19.1 ± 1.8 µM, mean ± standard deviation [SD]) that was unable to turn over nitrite are in good agreement with the levels required for full restoration of growth of the *nos* mutant, suggesting that most of the NO· produced by NOS in the wild-type strain was converted to nitrite under aerobic conditions.

### Nitrite enhances pyruvate dehydrogenase activity.

**FIG 4:**
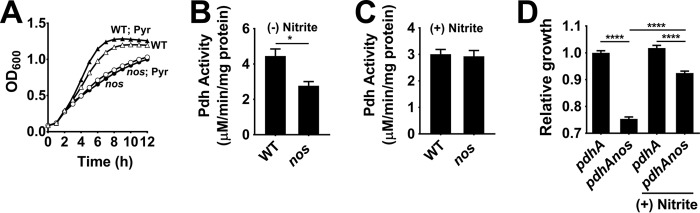
Reduced Pdh activity partly accounts for altered growth of the *nos* mutant. (A) Growth of the wild-type (WT) strain and *nos* mutant following pyruvate (5 mM) supplementation (means ± SEM, *n* = 5). (B) Pdh activity of the WT strain and *nos* mutant was measured following 6 h of growth in TSB with 14 mM glucose (means ± SEM, *n* = 7, Student’s *t* test; *, *P* < 0.05). (C) Pdh activity following nitrite supplementation (means ± SEM, *n* = 3). (D) AUCs of the *pdhA* and *pdhA nos* double mutants were calculated following growth in the presence or absence of 0.5 mM nitrite (see Fig. S5C). Relative growth levels were determined by comparing the AUC values of all mutants to that of the *pdhA* single mutant (means ± SEM, *n* = 6, one-way ANOVA, Tukey’s posttest; ****, *P* < 0.00005).

Although nitrite is uniquely capable of restoring growth of the *nos* mutant, it is not clear how nitrite accomplishes this task. Since we earlier ruled out perturbations in the TCA cycle as the cause of the growth defect in the *nos* mutant, we looked for targets upstream of the TCA cycle. Analysis of ^13^C_6_-labeled glucose distributions by NMR revealed a decrease in acetyl-coenzyme A (acetyl-CoA) levels in the *nos* mutant, suggesting a decrease in pyruvate dehydrogenase (Pdh) activity (Fig. 1B and C). To test this hypothesis, we initially monitored growth of the wild-type strain and *nos* mutant in TSB containing pyruvate rather than glucose as the primary carbon source. Consistent with decreased Pdh activity in the *nos* mutant, addition of pyruvate did not affect its growth (Fig. 4A). In contrast, both the growth rate and yield of the wild-type strain were increased (Fig. 4A). Further, direct estimation of Pdh activity confirmed a decrease in its activity in the *nos* mutant (Fig. 4B) which could be restored to wild-type levels following nitrite supplementation (Fig. 4C).

We reasoned that if the observed decrease in Pdh activity was the sole reason for the growth defect in the *nos* mutant, then the growth defect associated with the *pdh* mutant should phenocopy that of the *pdhA nos* double mutant. Instead, our analysis revealed that growth defects associated with *nos* and *pdhA* mutations were additive in the *pdhA nos* double mutant and not epistatic (Fig. 4D), suggesting that Pdh was not the primary target of NOS-mediated nitrite. Interestingly, nitrite supplementation restored growth of the *pdhA nos* double mutant only partially (Fig. 4D). Collectively, these results suggest that the ability of nitrite to increase Pdh activity may account only partly for the enhanced growth of the *nos* mutant following nitrite stimulation.

### SrrAB signaling compensates for growth following *nos* inactivation.

**FIG 5:**
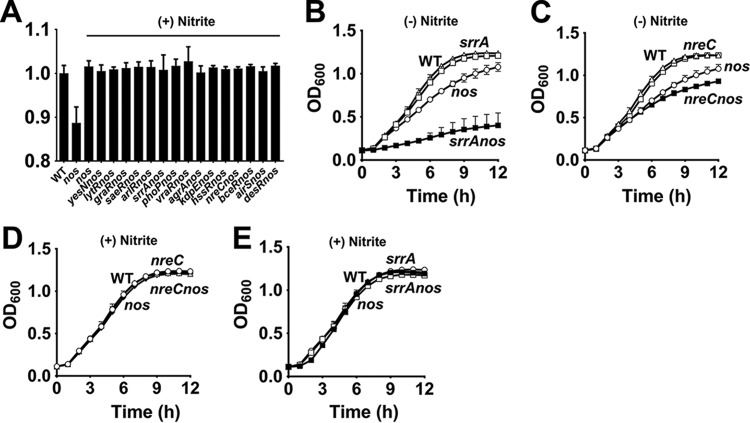
SrrA and NreC partially compensate for the growth defect in the *nos* mutant. (A) AUCs of the *nos* single mutant and 16 TCS *nos* double mutants were calculated following growth in the presence of 0.5 mM nitrite, and data are plotted relative to the AUC of the wild-type strain (means ± SEM, *n* = 3). (B and C) Growth of *srrA nos* (B) and *nreC nos* (C) double mutants relative to a *nos* single mutant (means ± SEM, *n* = 3). (D and E) Effect of nitrite supplementation (0.5 mM) on *nreC nos* (D) and *srrA nos* (E) double mutants relative to the wild-type strain (means ± SEM, *n* = 3).

Since extracellular nitrite could stimulate growth of the *nos* mutant at micromolar concentrations, we suspected that a nitrite-dependent signal transduction event mediated through an *S. aureus* two-component system (TCS) may be involved. To test this hypothesis, we transduced mutations of all nonessential TCSs available from the Nebraska Transposon Mutant Library (NTML) into the *nos* mutant and assessed whether supplementation of nitrite restored the growth defect associated with the *nos* mutation. We observed that nitrite was still capable of enhancing growth of the double mutants (Fig. 5A) relative to the *nos* mutant. This suggests that none of the nonessential *S. aureus* TCSs tested are involved in activating growth of the *nos* mutant in response to nitrite.

Surprisingly, we observed that inactivation of SrrAB and NreBC TCSs were detrimental to growth of the *nos* mutant. Whereas mutation of *srrA* dramatically decreased exponential-phase growth (Fig. 5B), an *nreC* mutation had a modest effect on stationary-phase yields of the *nos* mutant (Fig. 5C). Interestingly, supplementation of nitrite restored growth of the *srrA nos* and *nreC nos* double mutants, clearly indicating that nitrite did not mediate its growth-enhancing effects through either SrrA or NreC (Fig. 5D and E). Rather, these observations suggest that both SrrA and NreC are involved in regulating pathways that minimize growth defect of the *nos* mutant, albeit to various extents.

Since both SrrAB and NOS-dependent NO· production have previously been implicated in resistance to ROS (13), we hypothesized that the observed growth defect of the *srrA nos* mutant relative to the wild-type strain resulted from elevated endogenous ROS production. To test this hypothesis, we quantified endogenous ROS production in the wild type and the *srrA*, *nos*, and *srrA nos* mutants by electron paramagnetic resonance (EPR) spectroscopy (Fig. S6). No evidence of endogenous ROS production was observed in any of these mutants relative to the wild-type strain at the late-exponential-growth phase (6 h) despite a decrease in the growth rate of the *srrA nos* mutant relative to that of the wild-type strain. Thus, it is unlikely that endogenous ROS contributed to the growth defect observed in the *srrA nos* mutant. However, a modest increase in endogenous ROS production was detected in the *srrA nos* double mutant in the stationary phase (24 h) (Fig. S6). The observed level of ROS production was readily reversed following growth in the presence of nitrite. This suggested that ROS production in the *srrA nos* mutant resulted from a lack of NOS-dependent nitrite production rather than specific resistance mechanisms previously attributed to NO· (4).

10.1128/mBio.00887-17.7 FIG S6  EPR analysis. Download FIG S6, TIF file, 2 MB. Copyright © 2017 Chaudhari et al. 2017 Chaudhari et al. This content is distributed under the terms of the Creative Commons Attribution 4.0 International license.

### Nitrite activates respiration.

Given that the SrrAB TCS is thought to sense deficiencies in respiration and upregulate compensatory mechanisms (14), it is conceivable that *nos* activity can regulate respiration through nitrite production. Thus, respiration may be defective in a *nos* mutant. Consistent with this argument, NMR analysis indicated an increase in NADH/NAD^+^ ratio of the *nos* mutant compared to the wild-type strain (Fig. 1B and C), despite a decrease in TCA cycle and Pdh activities in the *nos* mutant (Fig. 1B to G and 4B). An increased NADH/NAD^+^ redox ratio could result from reduced functionality of the NADH dehydrogenase (encoded by *ndhA*) associated with the electron transport chain (ETC). To test this hypothesis, we utilized the *ndhA* mutant and its isogenic *ndhA nos* double mutant. Consistent with effects of an altered redox state, the *ndhA* mutant exhibited a growth defect (Fig. S7A). However, our analysis also revealed that the magnitude of the growth defect in the *ndhA nos* double mutant was greater than that seen with the *nos* mutant alone (Fig. S7A), suggesting that *nos* and *ndhA* potentially affected growth through distinct pathways. Further, the presence of nitrite fully restored the *nos-*dependent growth defect in the double mutant to the levels observed in the *ndhA* single mutant (Fig. S7B), confirming that *ndhA* was not the target of nitrite derived from NOS activity.

10.1128/mBio.00887-17.8 FIG S7  The growth defect of the *nos* mutant does not result from deficiencies in most components of the electron transport chain. Download FIG S7, TIF file, 2.3 MB. Copyright © 2017 Chaudhari et al. 2017 Chaudhari et al. This content is distributed under the terms of the Creative Commons Attribution 4.0 International license.

**FIG 6:**
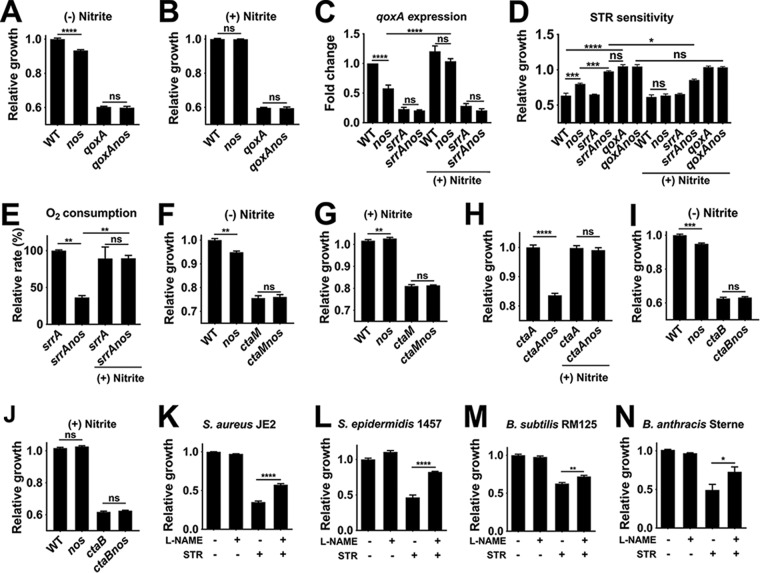
Nitrite derived from NOS activates cytochrome *bo*. (A and B) AUCs of the *qoxA* and *qoxA nos* mutants relative to the wild-type strain (relative growth) were determined in the absence (A) or presence (B) of 0.5 mM nitrite (means ± SEM, *n* = 8, one-way ANOVA, Tukey’s posttest). Corresponding growth curves for these mutants are depicted in Fig. S8A and B, respectively. (C) *qoxA* transcription was assessed by quantitative real-time PCR using *sigA* as an internal control (means ± SEM, *n* = 3, one-way ANOVA, Tukey’s posttest). (D) Ratios of the AUCs of all strains following streptomycin (STR) challenge (6.25 µg/ml) to that of the corresponding untreated controls (relative growth) were determined in the presence (0.5 mM) or absence of nitrite in 50%-diluted TSB containing 7 mM glucose (means ± SEM, *n* = 6, one-way ANOVA, Tukey’s posttest). (E) The rate of oxygen consumption relative to the *srrA* single mutant was determined using a MitoXpress-Xtra oxygen-sensitive phosphorescent probe (Luxcel Biosciences) (means ± SEM, *n* = 3, one-way ANOVA, Tukey’s posttest). (F and G) AUCs of the *ctaM* and *ctaM nos* mutants relative to the wild-type strain were determined following growth in the absence (F) or presence (G) of 0.5 mM nitrite (means ± SEM, *n* = 3, one-way ANOVA, Tukey’s posttest). Corresponding growth curves for these strains are depicted in Fig. S8C and D, respectively. (H) AUCs of the *ctaA* and *ctaA nos* mutants grown in the presence of nitrite relative to the untreated *ctaA* mutant (means ± SEM, *n* = 3, one-way ANOVA, Tukey’s posttest). Corresponding growth curves for these strains are depicted in Fig. S8E. (I and J) AUCs of the *ctaB* and *ctaB nos* mutants relative to the wild-type strain were determined following growth in the absence (I) or presence (J) of 0.5 mM nitrite (means ± SEM, *n* = 3, one-way ANOVA, Tukey’s posttest). Corresponding growth curves for these mutants are depicted in Fig. S8F and G, respectively. (K to N) AUCs of *S. aureus* JE2 (K), *S. epidermidis* 1457 (L), *B. subtilis* RM125 (M), and *B. anthracis* Sterne (N) grown in the presence or absence of l-NAME (2 mM, NOS inhibitor) and streptomycin were determined and compared to those measured for their respective untreated wild-type controls (means ± SEM, *n* = 3, one-way ANOVA, Tukey’s posttest). Corresponding growth curves for these mutants are depicted in Fig. S8H to K, respectively. The streptomycin concentrations used were as follows: 6.25 µg/ml (*S. aureus* and *B. subtilis*), 0.781 µg/ml (*S. epidermidis*), and 0.195 µg/ml (*B. anthracis*). n.s., not significant; *, *P* < 0.05, **, *P* < 0.005; ***, *P* < 0.0005; ****, *P* < 0.00005.

It is conceivable that menaquinone levels may be affected in the *nos* mutant due to decreased levels of intracellular glutamate precursor pools (15). However, assays wherein menaquinone was supplemented to media did not rescue the growth defect observed in the *nos* mutant (Fig. S7C). Finally, we tested if *S. aureus* cytochromes (quinol oxidase and CydBD encoded by the *qoxABCD* and *cydAB* operons, respectively) were the target of nitrite. Inactivation of cytochromes can result in growth defects (16, 17). If the growth defect associated with a *nos* mutation resulted from its ability to modulate cytochrome activity, we would expect that *qoxA nos* and *cydA nos* double mutants would not have an additive growth defect relative to their respective single mutants. Furthermore, supplementation of nitrite during growth of *cydA nos* and *qoxA nos* double mutants should prevent restoration of growth. Upon testing this hypothesis, our results revealed that CydAB was not the target of nitrite, as its inactivation did not result in a growth defect and nitrite could rescue the growth defect in the *cydA nos* double mutant to the levels observed in the wild-type strain (Fig. S7D and E). Interestingly, although the *qoxA* mutation conferred an enhanced growth defect compared to the *nos* mutant (Fig. 6A), the defects were not additive in the *qoxA nos* double mutant, suggesting that *qoxA* is epistatic with respect to *nos* (Fig. 6A). Consistent with this interpretation, the growth defect associated with the *qoxA nos* double mutant could not be restored after nitrite supplementation (Fig. 6B), indicating that the *S. aureus* quinol oxidase is the likely target of nitrite derived from NOS activity.

10.1128/mBio.00887-17.9 FIG S8  Nitrite derived from NOS targets *S. aureus* quinol oxidase. Download FIG S8, TIF file, 2.7 MB. Copyright © 2017 Chaudhari et al. 2017 Chaudhari et al. This content is distributed under the terms of the Creative Commons Attribution 4.0 International license.

Since it is not evident how nitrite may affect *qoxA* activity, we initially tested whether nitrite activates *qoxA* expression in the *nos* mutant. As the compensatory effects of SrrAB, a known activator of *qoxA* transcription (14), could mask any effects nitrite has on its expression, we assayed *qoxA* transcription by quantitative real-time PCR (qRT-PCR) in the *srrA* and *srrA nos* mutants. Our results did not reveal any significant increase in *qoxA* transcription after addition of nitrite in the *srrA nos* double mutant (Fig. 6C) despite nitrite being able to restore growth of the *srrA nos* double mutant (Fig. 5E). This suggests that nitrite potentially affects quinol oxidase activity rather than its transcription. To confirm this hypothesis, we designed a bioassay that directly tested the functionality of the *S. aureus* quinol oxidase. In *Bacillus subtilis*, streptomycin sensitivity is specifically dependent on quinol oxidase activity (18, 19). *B. subtilis* quinol oxidase mutants are significantly resistant to streptomycin (18). We confirmed a similar resistance phenotype in a *S. aureus qoxA* mutant challenged with streptomycin (Fig. 6D). We reasoned that if NOS was required to activate QoxA activity, an *S. aureus nos* mutant should exhibit resistance toward streptomycin relative to the wild-type strain. This was indeed the case, although the resistance to streptomycin was relatively modest in the *nos* mutant compared to a *qoxA* mutant (Fig. 6D). The reduced resistance in the *nos* mutant was primarily due to activity of SrrAB, as the *srrA nos* double mutant exhibited streptomycin resistance comparable to that shown by the *qoxA* single mutant. Further confirming a role for *nos* in activation of quinol oxidase function, we observed that an *srrA nos* double mutant was severely compromised in respiration relative to the *srrA* single mutant as assessed by its ability to consume oxygen (Fig. 6E). Significantly, both phenotypes (decreased oxygen consumption and streptomycin resistance) associated with the *srrA nos* double mutant could be complemented by nitrite supplementation (Fig. 6D and E), thus bypassing the requirement of NOS. Further supporting a role for *nos-*derived nitrite in activating respiration, the growth defect of a *ctaM nos* double mutant could not be restored to a magnitude of growth similar to that observed in the *nos* single mutant even after supplementation of nitrite in the media (Fig. 6F and G). As *ctaM* is thought to affect integration of heme A and heme O into quinol oxidases (17), our results collectively suggest that nitrite excreted due to NOS activity directly activates respiration by affecting quinol oxidase function.

Since quinol oxidase houses both heme A and heme O, resulting in cytochrome *aa*_3_ and cytochrome *bo*, respectively, we next attempted to determine which of these cytochromes are targeted by *nos-*derived nitrite. Inactivation of *ctaA* abrogates heme A, whereas mutation of *ctaB* abolishes both heme O and heme A biosynthesis (17). However, relative to *ctaA* and *ctaB* single mutants, addition of nitrite rescued growth of the *ctaA nos* mutant (Fig. 6H) but not that of a *ctaB nos* double mutant (Fig. 6I and J), respectively. This suggests that nitrite-mediated activation of respiration is potentially due to its effects on the cytochrome *bo* oxidase rather than cytochrome *aa*_3_. Finally, to test whether the ability of NOS to activate quinol oxidase function is conserved in bacteria, we inhibited endogenous NOS activity by the addition of a competitive inhibitor, L-N-nitroarginine methyl ester (l-NAME), and tested for resistance to streptomycin. Interestingly, the effect of l-NAME on bacterial NOS activity was not evident in itself as none of these strains displayed a growth defect similar to that seen with the *S. aureus nos* mutant. The reason for this result is currently unknown, but it may reflect incomplete inhibition of bacterial NOS by l-NAME. However, all strains, including *S. epidermidis* (Fig. 6L), *B. subtilis* (Fig. 6M), and *B. anthracis* (Fig. 6N), displayed an increase in resistance to streptomycin following treatment with l-NAME similar to that observed in *S. aureus* (Fig. 6K), suggesting that the effect of NOS activity on respiration is conserved across different bacterial species.

## DISCUSSION

NO· is a potent inhibitor of aerobic respiration and central metabolism due to its ability to react with heme centers, protein thiols, and Fe-S clusters in enzymes (8, 20). Consequently, it is unclear how bacteria that harbor NOS and endogenously produce NO· avoid its autotoxic effects. Furthermore, multiple studies have indicated that NOS activity promotes staphylococcal colonization and pathogenesis *in vivo* rather than impairing growth (6, 7). A recent study attempted to explain this apparent contradiction by suggesting that conversion of endogenously generated NO· to nitrate by the *S. aureus* flavohemoglobin, Hmp, not only would be an efficient process of NO· detoxification but would also promote nitrate reductase-mediated respiration under microaerobic conditions (7). Although such a mechanism seems likely, the relative contribution of NOS-dependent nitrate respiration to the overall growth of *S. aureus*, barring specialized growth conditions (for, e.g., following daptomycin challenge [7]), is yet to be fully ascertained, especially given the low concentrations of NO· generated through NOS activity. In this study, we demonstrated that micromolar concentrations of nitrite, rather than nitrate *per se*, resulting from NOS activity represent the predominant physiological effector of NOS function. Thus, *S. aureus* not only detoxifies NO· to nitrate and nitrite but also activates cytochrome *bo* oxidase-dependent respiration by repurposing NO· to nitrite.

Much of our current understanding of cytochrome *bo* oxidases is derived from studies carried out in *Escherichia coli*. Prokaryotic cytochrome *bo* oxidases are part of the heme copper oxidase superfamily and usually contains four subunits (21, 22). Of these, subunit 1 houses the metal redox centers, which include the six-coordinate low spin heme *b* and the heme *o*-copper (Cu_B_) binuclear center that reduces oxygen to water (23). The cytochrome *bo* oxidase also translocates protons across the cytoplasmic membrane, thus contributing to a membrane potential (23). Previously, it was shown that nitrites, like nitric oxide, can bind cytochrome heme centers and inactivate respiration (24). However, it must be noted that cytochrome *bo* oxidase has a low affinity for nitrite and thus millimolar levels, rather than the micromolar levels produced by staphylococcal NOS, would be required to inactivate it (24, 25). Furthermore, continuous depletion of nitrite due to nitrite reductase activity would also prevent accumulation of inhibitory nitrite concentrations in the immediate environment of cells. Surprisingly, our results suggest that low concentrations of nitrite generated due to NOS activity can activate respiration by affecting cytochrome *bo* oxidase. Several lines of evidence confirm this. First, addition of nitrite increased the rate of respiration of an *srrA nos* double mutant that exhibited low basal levels of respiration compared to the *srrA* or *nos* single mutants. Second, both *nos* and *srrA nos* mutations promoted streptomycin resistance in *S. aureus* and nitrite supplementation reversed this phenotype in both mutants. Since it was previously demonstrated in *Bacillus subtilis* (18, 19) and confirmed presently in *S. aureus* that streptomycin resistance is inversely proportional to quinol oxidase activity, this lends credible support to the conclusion that nitrite activates cytochrome oxidase function. Finally, while nitrite supplementation could rescue growth of the *nos* mutant to wild-type levels, it failed to do so in *qoxA nos* and *ctaB nos* double mutants, clearly demonstrating that nitrite-mediated activation of cytochrome *bo* oxidase is necessary for optimal growth.

Indirect evidence that points to NOS modulating cytochrome *bo* oxidase function may also be surmised. For instance, the inhibitory effect on pyruvate oxidation and TCA cycle activity upon *nos* mutation may be attributed to decreased quinol oxidase function and altered redox ratios. Similar effects were observed in *B. subtilis* when quinol oxidase function was inhibited (26). In a separate study, mutation of staphylococcal *nos* resulted in increased heme sensitivity (27). This may now be partly explained by the defective quinol oxidase activity in the *nos* mutant which is reported to sensitize cells to heme (27). Collectively, these data confirm a role for NOS in modulating cytochrome *bo* oxidase activity through the generation of nitrite.

How does nitrite activate *S. aureus* cytochrome *bo* oxidase-dependent respiration? Nitrite may be required for *qox* expression. In support of this hypothesis, quantitative real-time PCR (qRT-PCR) analysis revealed a modest but significant decrease in *qox* expression following *nos* mutation which was reversed upon nitrite supplementation. However, this is unlikely to completely account for the observed differences in respiration since nitrite also activated respiration in an *srrA nos* double mutant independently of *qoxA* expression. Another possibility is that nitrite might prevent hydrogen sulfide (H_2_S)-mediated inhibition of cytochrome *bo* oxidase. H_2_S, a by-product of cysteine metabolism, may bind both the heme iron and copper present in cytochrome *bo* oxidase, thus inhibiting its activity (28, 29). NOS-dependent nitrite may directly scavenge H_2_S or may activate proteins involved in sulfide detoxification (30). In support of the latter hypothesis, it was previously observed that nitrite strongly activated the *cst* operon involved in sulfide detoxification (31, 32). Alternatively, a direct stimulatory effect on cytochrome *bo* oxidase activity by nitrite, despite the lack of a structural basis for such a hypothesis, cannot be ruled out. Irrespective of the mechanism of activation, cytochrome *bo* oxidase activity prevents metabolic dysfunction, presumably due to the maintenance of redox balance (26).

Although the evidence presented in this study clearly suggests that nitrite affects cytochrome *bo* oxidase activity in the *nos* mutant, we cannot fully exclude the possibility of a role for nitrite in promoting cytochrome *aa*_3_ activity. It is possible that the *nos* mutant may produce limited quantities of cytochrome *aa*_3_; hence a role for nitrite in stimulating cyt *aa*_3_ activity was not evident in our assays. Additional studies that define the cytochrome profile of the *nos* mutant relative to that of the wild-type strain are necessary to demonstrate whether the effects of nitrite are exclusive to cytochrome *bo* oxidase activity.

**FIG 7:**
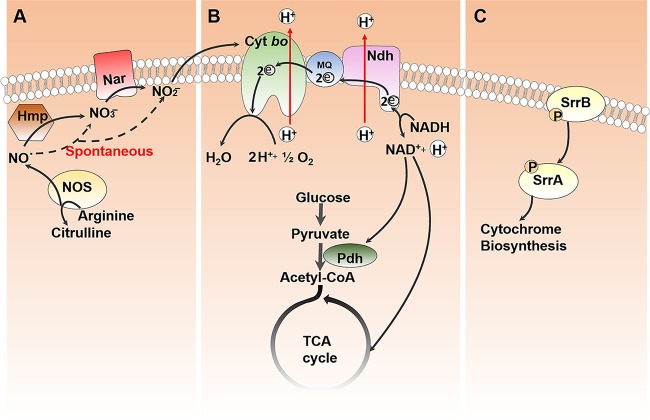
Proposed mechanism by which NOS activates respiration. (A) NO· generated due to NOS activity is rapidly decomposed into nitrite either spontaneously under aerobic conditions or through nitrate via Hmp and nitrate reductase activities. (B) Nitrite activates cytochrome *bo*-dependent respiration through an unknown mechanism and prevents metabolic dysfunction. (C) In the absence of NOS, SrrAB-dependent two-component signaling activates respiration and compensates for growth.

In conclusion, our results, along with those from other published studies, reveal a model (Fig. 7) wherein *nos* expression comes to prominence when cellular respiration is required to support growth. NO· generated due to NOS activity is rapidly converted to nitrite either spontaneously under aerobic conditions or via nitrate through Hmp activity (7). The generated nitrite activates cytochrome *bo* oxidase through a yet-unknown mechanism and promotes central metabolic functions. In the absence of NOS activity, SrrAB-mediated activation of respiration is dominant. While the NreBC two-component system also contributes to respiration, its effect is relatively minor relative to that of SrrAB. Consistent with a role for SrrAB in modulating respiration, its ability to regulate quinol oxidase expression was previously demonstrated (14). Given that inhibition of NOS activity affects quinol oxidase-dependent respiration in multiple bacterial species, a conserved role for NOS-dependent nitrite production in activating the terminal quinol oxidase is proposed.

## MATERIALS AND METHODS

### Bacterial strains, plasmids, and growth conditions.

*S. aureus* transposon mutants were obtained from the Nebraska Transposon Mutant Library (NTML). Retransduction of library mutants into wild-type JE2 and the respective mutant backgrounds was performed by ϕ11-mediated phage transduction (see Table S1 in the supplemental material). All mutants obtained from the NTML were confirmed by assays using the gene-specific primers listed in Table S1. Allelic exchanges to replace antibiotic cassettes in transposon mutants were performed essentially as described earlier (33). Unless otherwise mentioned, all *S. aureus* strains were grown aerobically in tryptic soy broth (TSB) supplemented with 14 mM glucose at 37°C and 245 rpm. Complementation of the JE2 *nos* mutant was carried out by expressing *nos* gene in *trans* using plasmid pSC27. This construct was created by PCR amplification of the *nos* gene, including its native promoter, from JE2 chromosomal DNA using primers listed in Table S1. The resulting fragment was cloned into an *E. coli-S. aureus* shuttle vector, pLI50, using a NEBuilder Hi-Fi DNA assembly cloning kit. Subsequently, pSC27 was transformed into RN4220 and later transduced into the *nos* mutant for phenotypic complementation. For expression and purification of NOS, plasmid pSC28 was constructed by cloning full-length *nos* into plasmid pET28a(+) and transformed into *E. coli* DH5*α* for maintenance. Expression of NOS was performed in *E. coli* BL21(DE3) following transformation with pSC28. Where appropriate, erythromycin, chloramphenicol, and kanamycin were supplemented in media at concentrations of 5, 10, and 30 µg/ml, respectively.

10.1128/mBio.00887-17.10 TABLE S1  List of strains and primers. Download TABLE S1, DOCX file, 0.02 MB. Copyright © 2017 Chaudhari et al. 2017 Chaudhari et al. This content is distributed under the terms of the Creative Commons Attribution 4.0 International license.

### NMR sample preparation.

Overnight cultures of strain JE2 and the isogenic *nos* mutant were inoculated to an optical density at 600 nm (OD_600_) of 0.06 units in TSB containing 14 mM ^13^C_6_-labeled glucose. Following 9 h of growth, culture volumes corresponding to a cell density of 40 OD_600_ units were rapidly harvested by centrifugation at 10,000 × *g* at 4°C, washed twice with cold saline solution (10 ml), and resuspended to a final volume of 1 ml in cold sterile water. Cultures were lysed by bead beating, and cellular debris and glass beads were pelleted by centrifugation. A supernatant volume of 0.8 ml was collected and stored on ice. The pellets were reextracted with an additional volume of 1 ml ice-cold sterile water and pooled with the previous extraction. Pooled fractions were flash-frozen using liquid nitrogen and stored at −80°C until further analysis.

### NMR data collection and analysis.

The collected supernatants were lyophilized and reconstructed in NMR buffer (potassium phosphate buffer in D_2_O, pH 7.4 [uncorrected], with 500 mM trimethylsilyl propanoic acid [TMSP] as an internal standard). 2D ^1^H-^13^C HSQC spectra were collected on a Bruker Avance III-HD 700-MHz spectrometer equipped with a quadrapole-resonance QCI-P cryoprobe (^1^H, ^13^C, ^15^N, and ^31^P), a SampleJet automated sample changer, Bruker ICON-NMR, and an automatic tuning and matching (ATM) unit. NMRPipe (34) and NMRViewJ (35) were used to process and analyze the collected spectra. The TMSP internal standard was used for chemical shift referencing and normalization of NMR peak intensities. NMR peaks from the 2D ^1^H-^13^C HSQC spectra were annotated by comparing the observed ^1^H and ^13^C chemical shifts to the metabolite reference data from the Platform for RIKEN Metabolomics (PRIMe) (36), Human Metabolome Database (HMDB) (37), Madison metabolomics Consortium Database (38), Metabominer (39), and BiomagResBank (BMRB) (40) with error tolerances of 0.08 ppm and 0.25 ppm for ^1^H and ^13^C chemical shifts, respectively. The relative intensity (i.e., concentration) of each metabolite was calculated by averaging the intensities of all NMR peaks unambiguously assigned to the metabolite.

### Growth assays.

For growth analysis, *S. aureus* cultures were grown aerobically at 37°C in 30 ml TSB supplemented with 14 mM glucose (in 250-ml Erlenmeyer flasks) for 24 h. Culture aliquots of 1 ml were collected hourly and used for optical density (OD_600_), pH, and metabolite analysis.

The effect of various compounds on *S. aureus* growth was determined by automated spectrophotometric analysis of culture densities (OD_600_) in a 96-well microtiter plate using a Tecan Infinite M200 spectrophotometer. Maximum aeration and a temperature of 37°C were selected in these assays. The media constituted 50% diluted TSB (vol/vol) without glucose. The OD_600_ was recorded every 30 min for 24 h. The various compounds that were tested include nitrite (0.5 mM), nitrate (5 mM), alpha-ketoglutarate (25 mM), succinate (25 mM), pyruvate (5 mM and 25 mM), urea (10 mM), cPTIO (200 µM), and menaquinone (25 µM). Streptomycin was added at concentrations of 6.25 µg/ml for *S. aureus* and *B. subtilis*, 0.781 µg/ml for *S. epidermidis*, and 0.195 µg/ml for *B. anthracis*.

Where appropriate, relative growth levels were calculated by determining the ratio of the area under the growth curve (AUC) of various strains challenged with different compounds to that of an untreated control (usually, the wild-type strain). However, depending on the experiment that was performed, these controls could differ and are noted appropriately in text. Statistical analysis was carried out by one-way analysis of variance (ANOVA) followed by Tukey’s postcomparison test or a Student’s *t* test, as appropriate. All experiments were carried out with at least three biological replicates, and significance was assessed at a *P* value of <0.05.

### Relative competitive fitness.

Overnight cultures of WT and mutant strains were diluted to an OD_600_ of 0.06 (1:1 ratio) in a single culture and allowed to compete for 24 h. Initial (time, *t* = 0 h) and final (*t* = 24 h) CFU counts per milliliter of WT and mutant strains were determined by plating appropriate dilutions on TSB agar plates with and without erythromycin at 5 µg/ml. Relative levels of competitive fitness (*W*) were calculated as follows: *W* = ln(*M*_f_/*M*_i_)/ln(WT_f_/WT_i_), where *M*_i_ and WT_i_ refer to mutant and wild-type bacterial colony counts (CFU per milliliter) at the initiation of the competition and *M*_f_ and WT_f_ to the counts at the finish, respectively.

### Metabolite analysis.

For metabolite analysis, aliquots of bacterial cultures (1 ml) were collected at the indicated respective time points and centrifuged at 16,000 × *g* for 3 min. The supernatants were collected and stored at −20°C for further analysis. High-performance liquid chromatography (HPLC) analysis was performed for quantitation of various metabolites as previously described (41). Briefly, supernatants were filtered through a 0.2-µm-pore-size nylon filter and then passed through a Bio-Rad Aminex HPX-87 column (Bio-Rad) to achieve metabolite separation. A sample volume of 5 µl was injected onto the column using an autosampler, and the column temperature was maintained at 65°C using a thermostatically controlled column compartment. Analytes were eluted isocratically with 0.005 M H_2_SO_4_ at 0.5 ml/min for 30 min. Chromatograms were integrated using Agilent ChemStation analysis software.

Determinations of levels of nitrite in culture supernatants were performed using a kit-based Griess assay (Thermo Fisher Scientific) per the manufacturer’s instructions.

### Expression and purification of NOS.

NOS was expressed as an N-terminal 6× His tag fusion protein. Protein expression was performed using a previously described autoinduction method (42). Briefly, 200 ml of autoinduction media containing 100 µg/ml d-aminolevulenic acid was inoculated with *E. coli* BL21(DE3) containing pSC28. The bacteria were grown aerobically (250 rpm) at 37°C for 18 h. Cells were recovered by centrifugation in a Sorvall centrifuge at 10,000 rpm for 10 min at 4°C. The cell pellet was resuspended in 1× phosphate-buffered saline (PBS) (20 ml) containing 1× cOmplete protease inhibitor cocktail (Roche Diagnostics) and passed thrice through an Emulsiflex at 10,000 lb/in^2^. Following centrifugation (30,000 × *g*, 30 min), the recombinant protein from the resulting supernatant fraction was purified by passage through a HisPur cobalt resin column, per the instructions of the manufacturer (Thermo Fisher Scientific). The expression and purification yields were monitored by SDS-PAGE.

### Western blot analysis.

*S. aureus* JE2 (WT) and the *nos* mutant were inoculated to a starting OD_600_ of 0.06 in TSB supplemented with 14 mM glucose (30 ml in a 250-ml Erlenmeyer flask, 245 rpm, 37°C) and grown for 24 h. Culture aliquots (2 ml) were collected at various time points (3 h, 6 h, 9 h, and 24 h) and centrifuged at 16,000 × *g* for 3 min. Cell pellets were then resuspended in 1× PBS and lysed by bead beating in an Omni Bead Ruptor 24-cell disrupter (Omni Internationals). Cell lysates were harvested and stored at −20°C until use. For detection of NOS, 50 µg of protein was separated on a 10% Tris-glycine SDS-polyacrylamide gel and Western blotting was performed using a 1:1,000 dilution of rabbit primary polyclonal antibody raised against staphylococcal NOS.

### Quantitative PCR (qPCR) analysis.

Quantitative reverse transcriptase PCR for detection of *qoxA* transcript levels was performed as previously described (43). Briefly, cDNA was synthesized from 500 ng of total RNA using a QuantiTect reverse-transcription kit (Qiagen) per the manufacturer’s instructions. The cDNA samples were then diluted 1:20 and subsequently used as the template for PCR. PCR amplification was done using PowerUp SYBR green master mix (Thermo Fisher Scientific) following the manufacturer’s instructions. The relative transcript levels were calculated using the comparative threshold cycle (*C*_*T*_) method and normalized to the amount of housekeeping sigma factor A (*sigA*) transcripts present in the RNA samples.

### Oxygen consumption.

*S. aureus* cultures were grown aerobically at 37°C in 25 ml TSB supplemented with 14 mM glucose for 24 h. After 6 h of growth, samples were collected and diluted to an OD_600_ of 0.1 units in fresh TSB supplemented with 14 mM glucose. Relative oxygen consumption rates were determined for a period of 30 min by using a MitoXpress oxygen-sensitive probe (Luxcel Biosciences) per the manufacturer’s instructions. Data are represented as percent oxygen consumption relative to *srrA* mutant strain results.

### Pyruvate dehydrogenase (Pdh) assay.

*S. aureus* cultures were grown aerobically at 37°C in 25 ml TSB supplemented with 14 mM glucose. Following 6 h of growth, cells were harvested and lysed and supernatants stored on ice until the assay was performed. The Pdh assay was performed as described earlier (44). Briefly, 200 µg of protein sample in a quartz cuvette was added to a master mix containing 2 mM sodium pyruvate, 1 mM MgCl_2_, 0.2 mM thiamine pyrophosphate (TPP), 0.5 mM 3-(4,5-dimethyl-2-thiazolyl)-2,5-diphenyl-2H-tetrazolium bromide (MTT), 6.5 mM phenazine methyl sulfate (PMS), and 50 mM potassium phosphate buffer (pH 7.1). Pdh activity was measured by examining the increase in absorbance at 560 nm for 5 min at 30-s intervals. The supernatant from an isogenic *pdhA* mutant served as a negative control.

### Electron paramagnetic resonance (EPR) spectroscopy.

EPR analysis was carried out as previously described (45). Briefly, 3-day-old stationary-phase bacterial cells were resuspended to an OD_600_ of 10 units in 1 ml of KDD buffer (Krebs-HEPES buffer [pH 7.4]; 99 mM NaCl, 4.69 mM KCl, 2.5 mM CaCl_2_, 1.2 mM MgSO_4_, 25 mM NaHCO_3_, 1.03 mM KH_2_PO_4_, 5.6 mM d-glucose, 20 mM HEPES, 5 µM diethyldithiocarbamic acid sodium salt [DETC], 25 µM deferoxamine). The bacterial samples were then mixed with 200 µM cell-permeative ROS-sensitive spin probe 1-hydroxy-3-methoxycarbonyl-2,2,5,5-tetramethylpyrrolidine (CMH; Noxygen Science Transfer and Diagnostics, Elzach, Germany) and incubated for 15 min at ambient temperature. EPR analysis was carried out using a Bruker e-scan EPR spectrometer with the following settings: field sweep width, 60.0 G; microwave frequency, 9.75 kHz; microwave power, 21.90 mW; modulation amplitude, 2.37 G; conversion time, 10.24 ms; time constant, 40.96 ms.
