# Supplementary material for: Nitrite Derived from Endogenous Bacterial Nitric Oxide Synthase Activity Promotes Aerobic Respiration
Source: mBio. 2017 Aug 1;8(4):e00887-17. doi: 10.1128/mBio.00887-17 (PMC5539425; doi:10.1128/mBio.00887-17)
Supplement: TEXT S1 [file mbo004173424s1.docx]

**Supplementary Text**

**Supplementary Figure legends**

**Figure S1. Representative 2D 1H-13C HSQC NMR spectrum of intracellular metabolites**. Metabolites were extracted from *S. aureus* wild-type. *S. aureus* strains were grown in the media supplemented with 13C labelled glucose and harvested at post-exponential phase. The two zoomed-in regions show visible intensity differences of metabolites acetate (#2) and ribose (#26) between wild-type (JE2) and NOS mutant. The annotation of unique peaks for some metabolites are described below. 1: Uridine; 2: Acetate; 3: Acetyl-aspartate; 4: Acetyl-CoA; 5: Acetyl-glucosamine; 6: Acetyl-glutamate; 7: Alanine; 8: Asparagine; 9: Aspartate; 10: Cystathionine; 11: D-Ala-D-ala; 12: dAMP; 13: dATP; 14: Erythrose 4-P; 15: Fructose 6-P; 16: Glucose-1-P; 17: Glutamate; 18: Glutamine; 19: GMP; 20: Histidine; 21: Lysine; 22: Methionine; 23: NADP+; 24: NADH; 25: Arginine; 26: Ribose; 27: Ribose 5-P; 28: Succinyl-CoA; 29: Threonine; 30: UDP; 31: Uracil; 32: NAD^+^; 33: Malate; 34: Leucine; 35: Deoxycytidine; 36: coA; 37: Phosphoglycerate.

**Figure S2. Complementation of the *nos* mutant.** Changes in growth (**A**) and pH (**B**) of the wild-type (WT) and *nos* mutant were monitored over 12 h (mean± SEM, n= 3). Expression of *nos* under its native promoter was performed *in trans* using plasmid pSC27. (**C)** Extracellular acetate concentrations were determined by HPLC analyses at 6 h of growth (mean ± SEM, n= 3, One-way ANOVA, Tukey’s post-test; *, *P*< 0.05, **, *P*< 0.005; ***, *P*< 0.0005).

**Figure S3. Growth defect of the *nos* mutant does not result from depletion of TCA cycle intermediates.** Growth of the wild-type (WT) and *nos* mutant following supplementation of (**A**) 5 mM citrate and (**C**) 25 mM succinate (mean ± SEM, n= 4). (**E**) Effect of α-Ketoglutarate on growth of the *citZ* mutant (mean ± SEM, n= 4). Consumption of (**B**) citrate and (**D**) succinate by *S. aureus* strains following 24 h of growth (representative trace, n= 4). Peaks represent metabolites from control samples that did not contain bacteria. cit, Citrate; suc, Succinate, KG, α-Ketoglutarate.

**Figure S4. Growth of *S. aureus* JE2 (WT) and *nos*, *sucA* and *citZ* mutants.** Growth was spectrophotometrically monitored (OD_600_) in TSB supplemented with 14mM glucose. Cultures were grown under aerobic conditions, 37˚C (mean ± SEM, n= 3).

**Figure S5. Growth analyses of JE2 (WT) and isogenic mutants.** Growth was spectrophotometrically measured (OD_600_) in 50% TSB (mean ± SEM, n= 6). (A) *nos* mutant relative to JE2 (± 10 mM ammonium chloride) (B) *nos*, *nirBnos* and *nirBnarDnos* mutants relative to JE2 (C) *pdhAnos* mutant relative to *pdhA* mutant (± 0.5 mM nitrite).

**Figure S6.** **EPR analysis.** Whole cell EPR spectroscopic analysis of JE2 (WT) and isogenic *nos*, *srrA*, *srrAnos* mutants were determined at 6h and 24h following growth in TSB supplemented with 14 mM glucose (A). In parallel, identical experiments were performed in media supplemented with 0.5mM nitrite (B). Cell densities were normalized prior to EPR analysis by measuring OD_600_ for all cultures at 6h and 24h (mean ± SEM, n= 3).

**Figure S7. Growth defect of the *nos* mutant does not result from deficiencies in most components of the electron transport chain. (A**) Growth (OD_600_) of NADH dehydrogenase (*ndhA*) single and *ndhAnos* double mutants were monitored over 12 h in 50% TSB without glucose (mean ± SEM, n= 4). (**B)** Growth of *ndhAnos* double mutant relative to *ndhA* single mutant in the presence or absence of 0.5 mM nitrite (mean ± SEM, n= 4, One-way ANOVA, Tukey’s post-test; ***, *P*< 0.0005). (**C)** Effect of menaquinone supplementation (25 µM) on wild-type (WT) and *nos* mutant (mean ± SEM, n= 6). (**D)** Growth of cytochrome *bd* oxidase (*cydA*) single and *cydAnos* double mutants were monitored over 12 h in 50% diluted TSB without glucose (mean ± SEM, n= 4). (**E)** Effect of nitrite (0.5 mM) supplementation on the growth of *cydAnos* double mutant and *cydA* mutant. Relative growth was determined by comparing the AUC of various samples relative to the untreated *cydA* single mutant (mean ± SEM, n= 8, One-way ANOVA, Tukey’s post-test; ****, *P*< 0.00005). n.s., not significant.

**Fig S8.** **Nitrite derived from NOS targets *S. aureus* quinol oxidase.** Growth of *qoxA*, *qoxAnos* (A-B), *ctaM*, *ctaMnos* (C-D), *ctaA*, *ctaAnos* (E), *ctaB*, *ctaBnos* (F-G) mutants relative to *S. aureus* JE2 (WT) was determined in presence or absence of 0.5 mM nitrite. *S. aureus* (H), *S. epidermidis* (I), *B. subtilis* (J) and *B. anthracis* (K) were grown in presence or absence of L-NAME (2mM) and streptomycin and compared to their respective untreated controls. Streptomycin concentrations are as follows: 6.25 ug/ml (*S. aureus* and *B. subtilis*), 0.781 ug/ml (*S. epidermidis*) and 0.195 ug/ml (*B. anthracis*) (mean ± SEM, n= 3).

**Supplementary Tables**

Table S1. List of strains and primers

**Supplementary References**

1. S. Ikawa, T. Shibata, T. Ando, and H. Saito, Mol Gen Genet 170:123-127, 1979.

2. L. Chandramohan, J. S. Ahn, K. E. Weaver, and K. W. Bayles, J Bacteriol 191:4103-4110, 2009.

3. D. Mack, N. Siemssen, and R. Laufs, Infect Immun 60:2048-2057, 1992.

4. B. N. Kreiswirth, S. Lofdahl, M. J. Betley, M. O'Reilly, P. M. Schlievert, M. S. Bergdoll, and R. P. Novick, Nature 305:709-712, 1983.

5. P. D. Fey, J. L. Endres, V. K. Yajjala, T. J. Widhelm, R. J. Boissy, J. L. Bose, and K. W. Bayles, MBio 4:e00537-00512, 2013.

6. C. Y. Lee, S. L. Buranen, and Z. H. Ye, Gene 103:101-105, 1991.
