# Supplementary figures and images for: Nitrite Derived from Endogenous Bacterial Nitric Oxide Synthase Activity Promotes Aerobic Respiration
Source: mBio. 2017 Aug 1;8(4):e00887-17. doi: 10.1128/mBio.00887-17 (PMC5539425; doi:10.1128/mBio.00887-17)

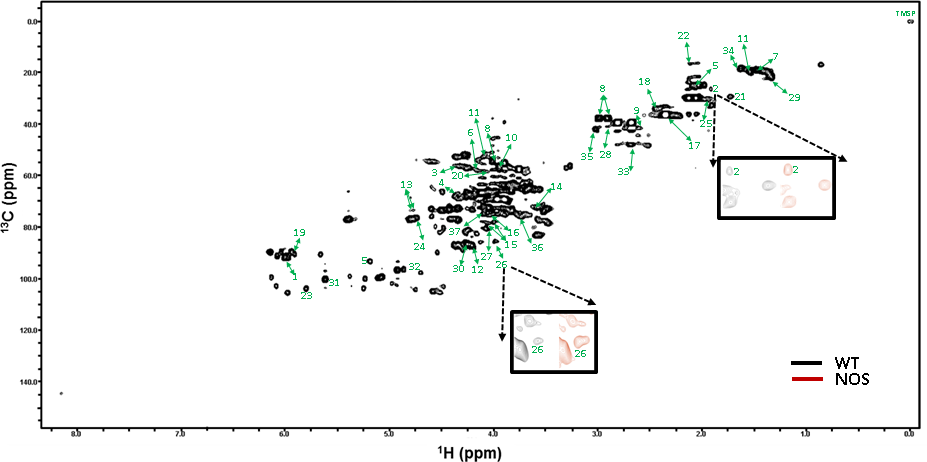

Supplement: FIG S1 [file mbo004173424sf1.tif]

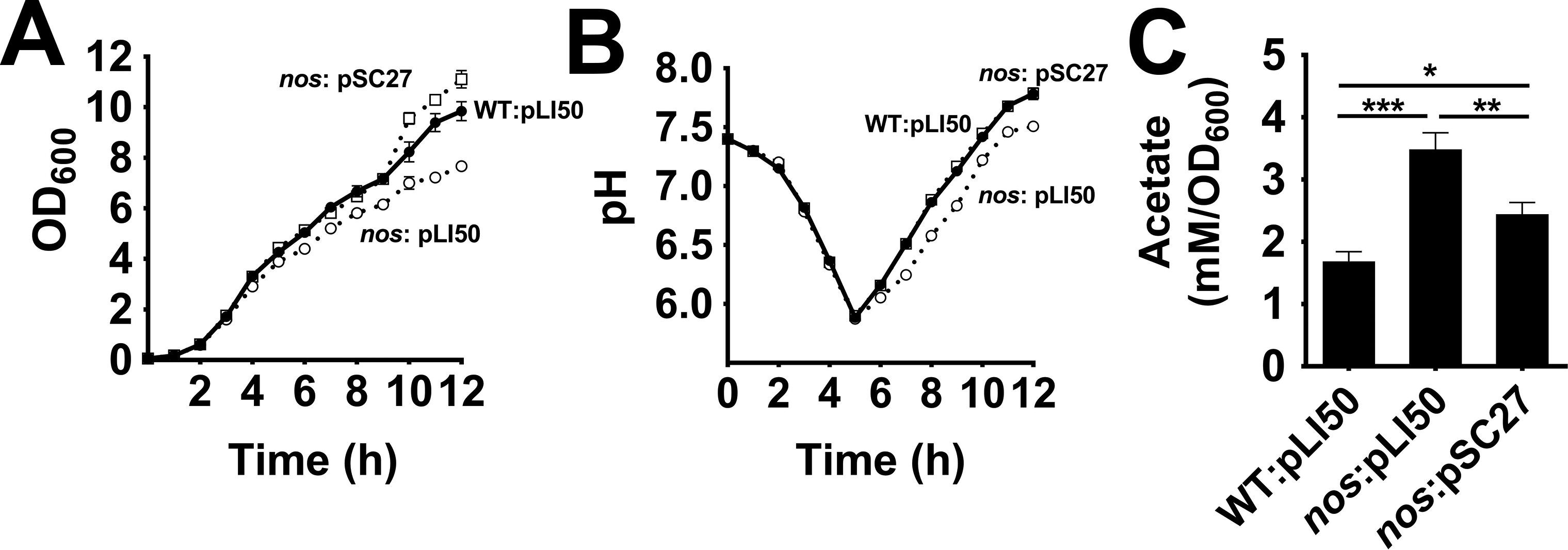

Supplement: FIG S2 [file mbo004173424sf2.tif]

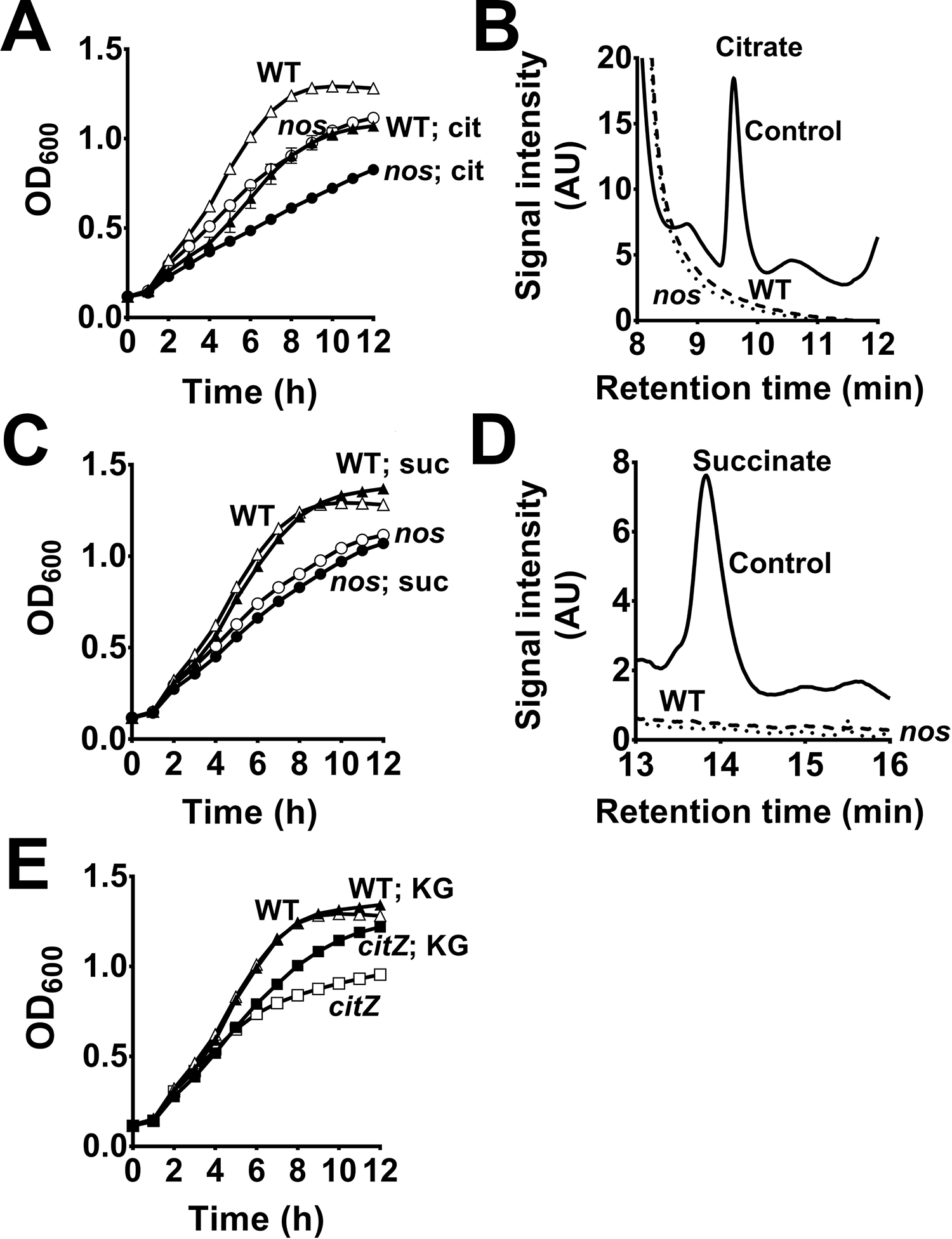

Supplement: FIG S3 [file mbo004173424sf3.tif]

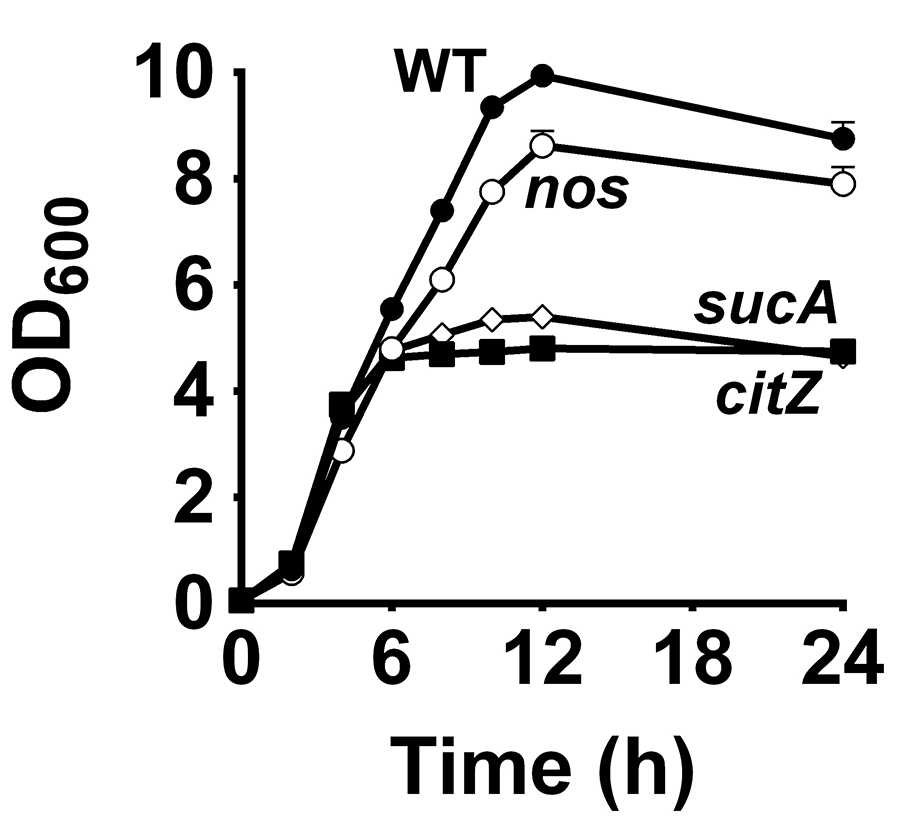

Supplement: FIG S4 [file mbo004173424sf4.tif]

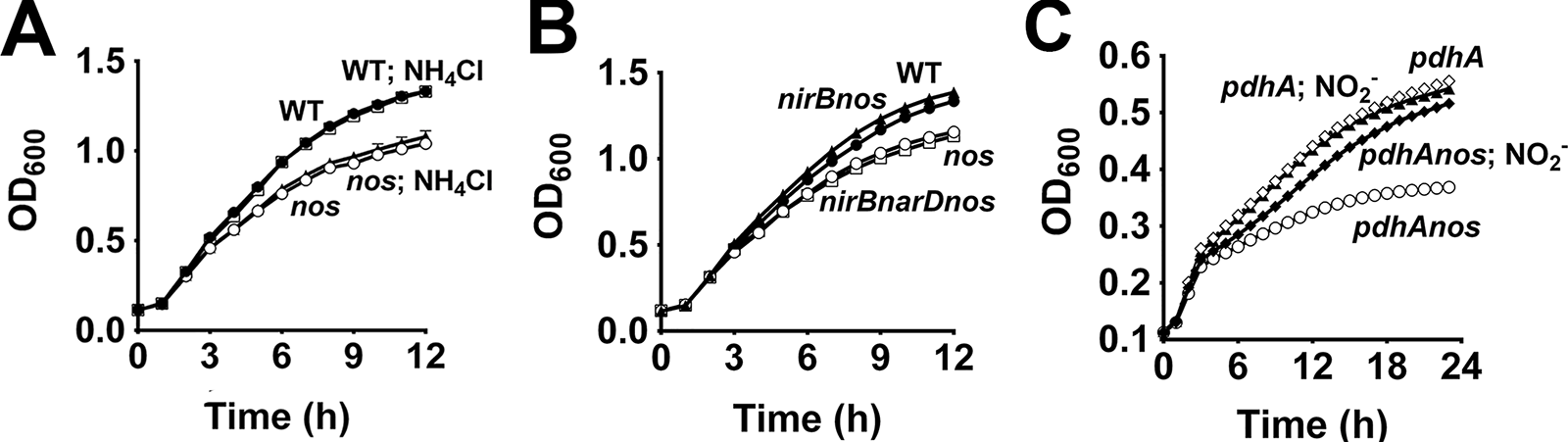

Supplement: FIG S5 [file mbo004173424sf5.tif]

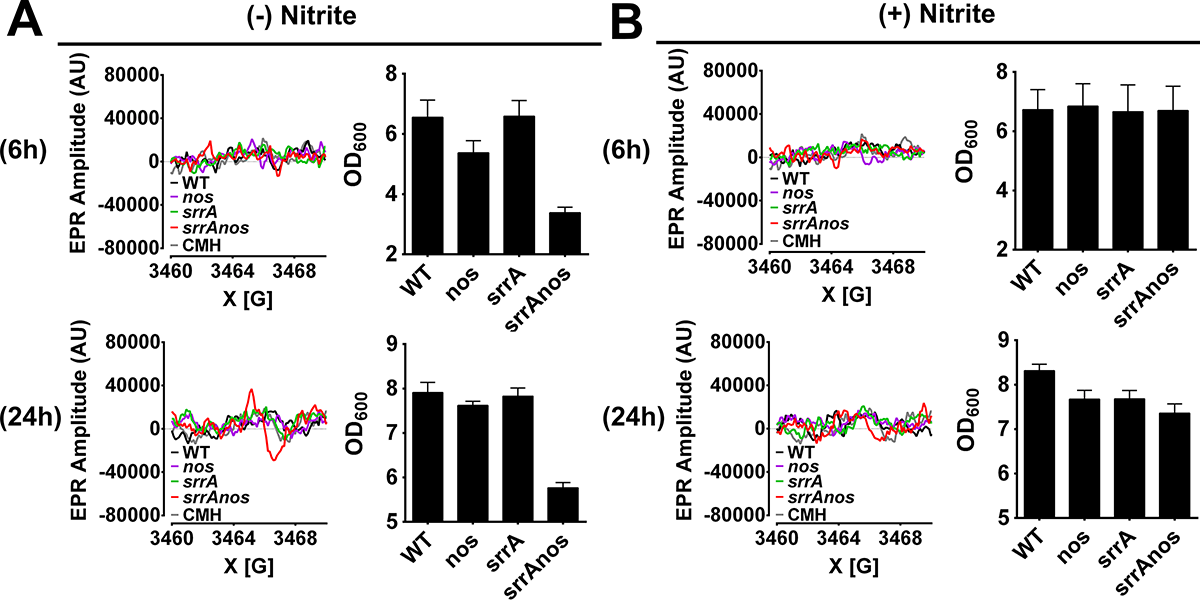

Supplement: FIG S6 [file mbo004173424sf6.tif]

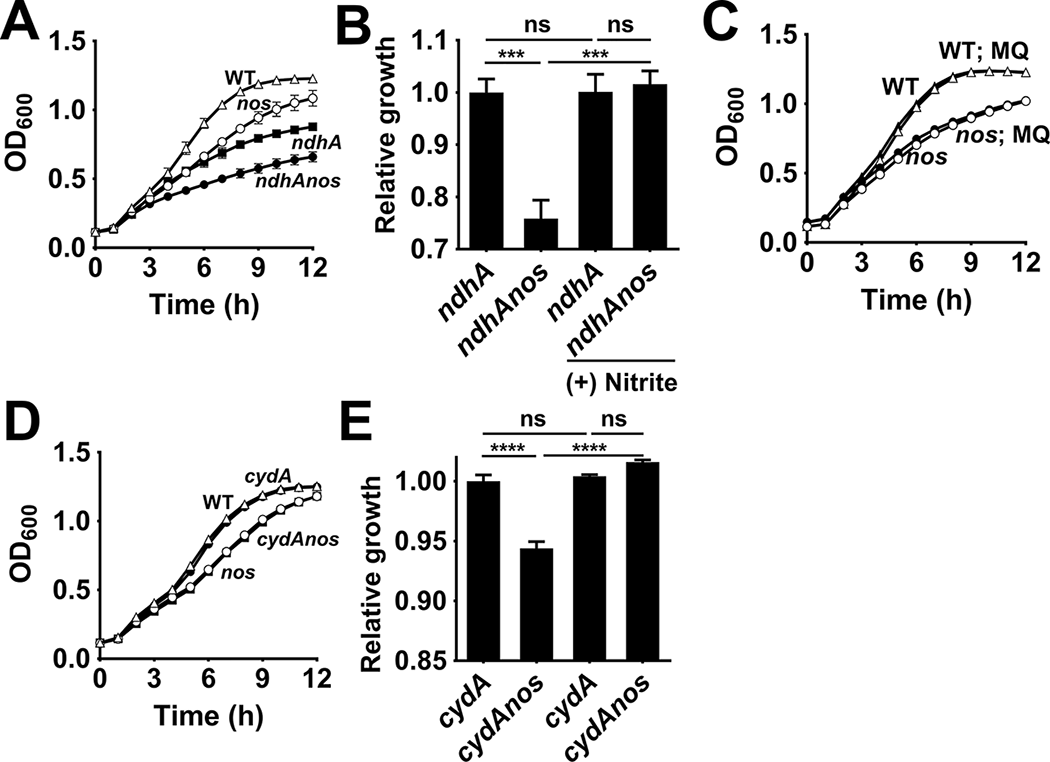

Supplement: FIG S7 [file mbo004173424sf7.tif]

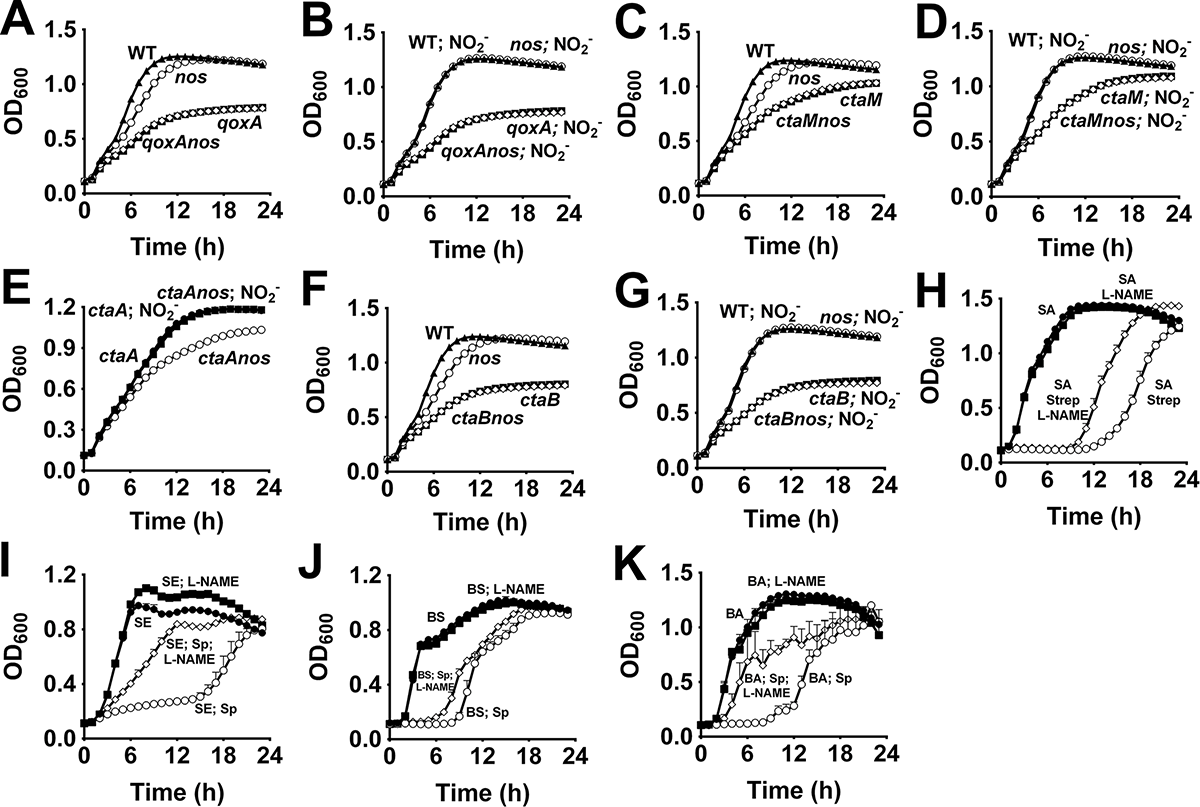

Supplement: FIG S8 [file mbo004173424sf8.tif]
