# Supplementary material for: Nitrite Derived from Endogenous Bacterial Nitric Oxide Synthase Activity Promotes Aerobic Respiration
Source: mBio. 2017 Aug 1;8(4):e00887-17. doi: 10.1128/mBio.00887-17 (PMC5539425; doi:10.1128/mBio.00887-17)
Supplement: TABLE S1 [file mbo004173424st1.docx]

| **Table S1** | **Strains and plasmids** | | | |  |  |
| --- | --- | --- | --- | --- | --- | --- |
|  | **Locus ID** | | **Description** | | **Source** |  |
| **Strains** | | | | | |  |
| *E. coli* Electro-Ten-Blue |  | | General plasmid maintenance strain | | Stratagene |  |
| *B. subtilis* RM125 |  | | *B. subtilis* Marburg 168 *hsrR*^-^ *hsrM*^-^ | | (Ikawa et al. 1979) |  |
| *B. antracis* Sterne |  | | B. anthracis (pXO1^+^ pXO2^−^) | | (Chandramohan et al. 2009) |  |
| *S. epidermidis* 1457 |  | | Clinical isolate (ica+ agr type II) | | (Mack et al. 1992) |  |
| *S. aureus* RN4220 |  | | Restriction deficient strain routinely used as a transformation intermediate | | (Kreiswirth et al. 1983) |  |
| *S. aureus* JE2 |  | | *S. aureus* USA300 LAC cured of all 3 native plasmids | | (Fey et al. 2013) |  |
| JE2 *nos* | SAUSA300_1895 | | *bursa aurealis* transposon mutant, Em^R^ | | NTML |  |
| JE2 *citZ* | SAUSA300_1641 | | *bursa aurealis* transposon mutant, Em^R^ | | NTML |  |
| JE2 *sucA* | SAUSA300_1306 | | *bursa aurealis* transposon mutant, Em^R^ | | NTML |  |
| JE2 *narA* | SAUSA300_2343 | | *bursa aurealis* transposon mutant, Em^R^ | | NTML |  |
| JE2 *nirB* | SAUSA300_2346 | | *bursa aurealis* transposon mutant, Em^R^ | | NTML |  |
| JE2 *pdhA* | SAUSA300_0993 | | *bursa aurealis* transposon mutant, Em^R^ | | NTML |  |
| JE2 *yesN* | SAUSA300_0217 | | *bursa aurealis* transposon mutant, Em^R^ | | NTML |  |
| JE2 *lytR* | SAUSA300_0255 | | *bursa aurealis* transposon mutant, Em^R^ | | NTML |  |
| JE2 *graR* | SAUSA300_0645 | | *bursa aurealis* transposon mutant, Em^R^ | | NTML |  |
| JE2 *saeR* | SAUSA300_0691 | | *bursa aurealis* transposon mutant, Em^R^ | | NTML |  |
| JE2 *arlR* | SAUSA300_1308 | | *bursa aurealis* transposon mutant, Em^R^ | | NTML |  |
| JE2 *srrA* | SAUSA300_1442 | | *bursa aurealis* transposon mutant, Em^R^ | | NTML |  |
| JE2 *phoP* | SAUSA300_1639 | | *bursa aurealis* transposon mutant, Em^R^ | | NTML |  |
| JE2 *vraR* | SAUSA300_1865 | | *bursa aurealis* transposon mutant, Em^R^ | | NTML |  |
| JE2 agrA | SAUSA300_1992 | | *bursa aurealis* transposon mutant, Em^R^ | | NTML |  |
| JE2 *kdpE* | SAUSA300_2036 | | *bursa aurealis* transposon mutant, Em^R^ | | NTML |  |
| JE2 *hssR* | SAUSA300_2308 | | *bursa aurealis* transposon mutant, Em^R^ | | NTML |  |
| JE2 *nreC* | SAUSA300_2337 | | *bursa aurealis* transposon mutant, Em^R^ | | NTML |  |
| JE2 *bceR* | SAUSA300_2559 | | *bursa aurealis* transposon mutant, Em^R^ | | NTML |  |
| JE2 *airS* | SAUSA300_1799 | | *bursa aurealis* transposon mutant, Em^R^ | | NTML |  |
| JE2 *desR* | SAUSA300_1220 | | *bursa aurealis* transposon mutant, Em^R^ | | NTML |  |
| JE2 *qoxA* | SAUSA300_0963 | | *bursa aurealis* transposon mutant, Em^R^ | | NTML |  |
| JE2 *ctaM* | SAUSA300_1017 | | *bursa aurealis* transposon mutant, Em^R^ | | NTML |  |
| JE2 *ctaA* | SAUSA300_1015 | | *bursa aurealis* transposon mutant, Em^R^ | | NTML |  |
| JE2 *ctaB* | SAUSA300_1016 | | *bursa aurealis* transposon mutant, Em^R^ | | NTML |  |
| JE2 *ndhA* | SAUSA300_0844 | | *bursa aurealis* transposon mutant, Em^R^ | | NTML |  |
| JE2 *cydA* | SAUSA300_0986 | | *bursa aurealis* transposon mutant, Em^R^ | | NTML |  |
| JE2 *narAnos* |  | | *bursa aurealis* transposon mutant, Em^R^, Tet^R^ | | This study |  |
| JE2 *nirBnos* |  | | *bursa aurealis* transposon mutant, Em^R^, Tet^R^ | | This study |  |
| JE2 *pdhAnos* |  | | *bursa aurealis* transposon mutant, Em^R^, Tet^R^ | | This study |  |
| JE2 *yesNnos* |  | | *bursa aurealis* transposon mutant, Em^R^, Tet^R^ | | This study |  |
| JE2 *lytRnos* |  | | *bursa aurealis* transposon mutant, Em^R^, Tet^R^ | | This study |  |
| JE2 *graRnos* |  | | *bursa aurealis* transposon mutant, Em^R^, Tet^R^ | | This study |  |
| JE2 *saeRnos* |  | | *bursa aurealis* transposon mutant, Em^R^, Tet^R^ | | This study |  |
| JE2 *arlRnos* |  | | *bursa aurealis* transposon mutant, Em^R^, Tet^R^ | | This study |  |
| JE2 *srrAnos* |  | | *bursa aurealis* transposon mutant, Em^R^, Tet^R^ | | This study |  |
| JE2 *phoPnos* |  | | *bursa aurealis* transposon mutant, Em^R^, Tet^R^ | | This study |  |
| JE2 *vraRnos* |  | | *bursa aurealis* transposon mutant, Em^R^, Tet^R^ | | This study |  |
| JE2 *agrAnos* |  | | *bursa aurealis* transposon mutant, Em^R^, Tet^R^ | | This study |  |
| JE2 *kdpEnos* |  | | *bursa aurealis* transposon mutant, Em^R^, Tet^R^ | | This study |  |
| JE2 *hssRnos* |  | | *bursa aurealis* transposon mutant, Em^R^, Tet^R^ | | This study |  |
| JE2 *nreCnos* |  | | *bursa aurealis* transposon mutant, Em^R^, Tet^R^ | | This study |  |
| JE2 *bceRnos* |  | | *bursa aurealis* transposon mutant, Em^R^, Tet^R^ | | This study |  |
| JE2 *airSnos* |  | | *bursa aurealis* transposon mutant, Em^R^, Tet^R^ | | This study |  |
| JE2 *desRnos* |  | | *bursa aurealis* transposon mutant, Em^R^, Tet^R^ | | This study |  |
| JE2 *qoxAnos* |  | | *bursa aurealis* transposon mutant, Em^R^, Tet^R^ | | This study |  |
| JE2 *ctaMnos* |  | | *bursa aurealis* transposon mutant, Em^R^, Tet^R^ | | This study |  |
| JE2 *ctaAnos* |  | | *bursa aurealis* transposon mutant, Em^R^, Tet^R^ | | This study |  |
| JE2 *ctaBnos* |  | | *bursa aurealis* transposon mutant, Em^R^, Tet^R^ | | This study |  |
| JE2 *ndhAnos* |  | | *bursa aurealis* transposon mutant, Em^R^, Tet^R^ | | This study |  |
| JE2 *cydAnos* |  | | *bursa aurealis* transposon mutant, Em^R^, Tet^R^ | | This study |  |
| JE2 *nirBnarDnos* |  | | *bursa aurealis* transposon mutant, Em^R^, Tet^R^ | | This study |  |
| **Plasmids** |  | |  | |  |  |
| pLI50 |  | | *E. coli*- *S. aureus* shuttle vector | | (Lee et al. 1991) |  |
| pSC27 |  | | pLI50 *nos* (under control of its native promoter) | | This study |  |
| pSC28 |  | | *E. coli* BL21(DE3) *nos* full length (N-terminal his tag) | | This study |  |
|  |  | |  | |  |  |
| **Primers** | | | | | | |
| **Gene** | | **Primer name** | | **Primer sequence (5’ - 3’)** | | |
| *nos* | | nos_F | | GAGGCTCAAGCTTTCATAGA | | |
|  | | nos_R | | GGCACTGGTTAGCATTTGAT | | |
| *narA* | | narA_F | | AATCGCAGCACAATTACTGT | | |
|  | | narA_R | | CTTCTTCCCAACGTTGACCC | | |
| *sucA* | | 1306_F | | GTGGAGGGTGTAAAATGACTAA | | |
|  | | 1306_R | | TTCGCAGCAATCGTAATAGTA | | |
| *citZ* | | 1641_F | | CAGCGGAGACTAAAATAAGTTC | | |
|  | | 1641_R | | CCCAATCTCAGATAACATCGTC | | |
| *nirB* | | nirB_F | | TGATTGGTAACGGTATGGCG | | |
|  | | nirB_R | | GCACTATACAAGTCACACCC | | |
| *pdhA* | | pdhA_F | | GTGACGGTGGTTCTTCACAA | | |
|  | | pdhA_R | | CTTCGACTCCTTCTCTTTGT | | |
| *yesN* | | yesN_F | | ATGGTTCACATGCACGACTC | | |
|  | | yesN_R | | GCCGTTCTGTGATTGTTG | | |
| *lytR* | | lytR_F | | TTGAATCGACATCGAGCGGT | | |
|  | | lytR_R | | TAGTTAAACCATTGTTGCACTTC | | |
| *graR* | | graR_F | | GGGTGATATGGATGCAAATACT | | |
|  | | graR_R | | CATCATCCCATAATGCAGTG | | |
| *saeR* | | saeR_F | | GATCGTGGATGATGAACAAGA | | |
|  | | saeR_R | | GGGACTTCGTGACCATTTAC | | |
| *desR* | | desR_F | | ACAGCGTGTACAACATTTAAATGG | | |
|  | | desR_R | | TCATCAATGTGGCGCTATATT | | |
| *arlR* | | arlR_F | | AGAAGATGAACAAAACTTAGCAAG | | |
|  | | arlR_R | | ATCACATACCCAACGCCACGAAC | | |
| *srrA* | | srrA_F | | GGTATGACCTGTATGTCGAACG | | |
|  | | srrA_R | | CTTCTTGATTATCAGCAAGTACG | | |
| *phoP* | | phoP_F | | ATGTCGCAAAAAGTGTTGGTAG | | |
|  | | phoP_R | | ATGCTCTCTCGTAATGACTC | | |
| *vraR* | | vraR_F | | CAGGTACACGTATCGAGGTGA | | |
|  | | varR_R | | TTCATATAACTCTGCGCGC | | |
| *agrA* | | agrA_F | | ATGGAAATTGCCCTCGCAAC | | |
|  | | agrA_R | | GACAATTCGCTCTTTCGAATC | | |
| *kdpE* | | kdpE_F | | CAGACTTTAACGAAGGAGACGT | | |
|  | | kdpE_R | | TTCATTTGGCGTCAAATGTAC | | |
| *hssR* | | hssR_F | | GGGAGTTTATAGCTATGGTGC | | |
|  | | hssR_R | | CCTTGTTCGGTAACGTCATC | | |
| *nreR* | | nreR_F | | TGATCACGCTGTTGTCCGTA | | |
|  | | nreR_R | | GCTTCTACTGTTTTCACAGATAC | | |
| *bceR* | | bceR_F | | CCGTCATTGCAGCAGGATAA | | |
|  | | bceR_R | | TTAATTTTTTACGCAGGCGC | | |
| *airS* | | airS_F | | TTGGAGCGATTTGATGGAAC | | |
|  | | airS_R | | TGTTTAGCGATGGATTCGTT | | |
| *cydA* | | cydA_F | | GCAACGATTGGTGTTGGTAT | | |
|  | | cydA_R | | TTGGCATGGGAAGATGTATC | | |
| *qoxA* | | qoxA_F | | CCAGTAGCAAGTAGTCAGAA | | |
|  | | qoxA_R | | TAGAGTTACGACCTCTGAACG | | |
| *ctaA* | | ctaA UP | | ACGAGATTACACAACAATTGAC | | |
|  | | ctaA DN | | ACGTGGATTACATAATTATCCCA | | |
| *ctaB* | | ctaB UP | | CTCTTGTCGTTAATTTGTCACA | | |
|  | | ctaB DN | | TGCACTAATGACAATACATGTC | | |
| *ctaM* | | ctaM UP | | AGTGATCTTTTTCGTGTTAGC | | |
|  | | ctaM DN | | ATCAACTGTCCGACTATTCTT | | |
| *ndhA* | | 0844_F | | GTAGCATTAGGTTTCGTTAGTG | | |
|  | | 0844_R | | GAATTCTTCAGTTGATTCACCG | | |
| pLI50 | | pLI50_F | | GAATTCTTGAAGACGAAAGGGCC | | |
|  | | pLI50_R | | GTCGACCTGCAGGCATGC | | |
| *nos* complement | | nos complement_F | | tttcgtcttcaagaattcGTTTCTATTAATTGCGCTTGAATC | | |
|  | | nos complement_R | | tgcctgcaggtcgacAGGGCCTAAATAATATAATTGC | | |
| *qoxA* RT PCR | | qoxA_RT_F | | ACGACAAATGGGTGAAAGAAG | | |
|  | | qoxA_RT_R | | CTGGATCAACAAACGCCATA | | |
| pET28a | | pET28a_fwd_N ter | | CAAAGCCCGAAAGGAAGC | | |
|  | | pET28a_rev_N ter | | GTGATGATGATGATGATGGC | | |
| NOS Full length protein | | NOS_N-ter_His_Tag_fwd | | agccatcatcatcatcatcacATGTTATTTAAAGAGGCTCAAG | | |
|  | | NOS_N-ter_His_Tag_rev | | ttcctttcgggctttgTTAATGATGGAAAGGGCAC | | |
|  | |  | |  | | |
